# The features analysis of hemoglobin expression on visual information transmission pathway in early stage of Alzheimer’s disease

**DOI:** 10.1038/s41598-024-64099-0

**Published:** 2024-07-08

**Authors:** Xuehui Li, Pan Tang, Xinping Pang, Xianghu Song, Jing Xiong, Lei Yu, Hui Liu, Chaoyang Pang

**Affiliations:** 1 College of Computer Science, Sichuan Normal University, Chengdu, 610101 China; 2 School of Information and Software Engineering, University of Electronic Science and Technology of China, Chengdu, 610054 China; 3 West China School of Basic Medical Sciences & Forensic Medicine, Sichuan University, Chengdu, 610041 China

**Keywords:** Alzheimer’s disease, Visual information transmission pathway, Hemoglobin, Computational biology and bioinformatics, Biomarkers, Medical research, Neurology, Mathematics and computing

## Abstract

Alzheimer's disease (AD) is a neurodegenerative disorder characterized primarily by cognitive impairment. The motivation of this paper is to explore the impact of the visual information transmission pathway (V–H pathway) on AD, and the following feature were observed: Hemoglobin expression on the V–H pathway becomes dysregulated as AD occurs so as to the pathway becomes dysfunctional. According to the feature, the following conclusion was proposed: As AD occurs, abnormal tau proteins penetrate bloodstream and arrive at the brain regions of the pathway. Then the tau proteins or other toxic substances attack hemoglobin molecules. Under the attack, hemoglobin expression becomes more dysregulated. The dysfunction of V–H pathway has an impact on early symptoms of AD, such as spatial recognition disorder and face recognition disorder.

## Introduction

Alzheimer's disease (AD) is a neurodegenerative disorder characterized primarily by cognitive impairment, particularly in advanced cognitive functions such as memory, learning, and thinking^1,2^. It is the most common form of dementia among the elderly, and its clinical course lacks effective curative medications. Once diagnosed, AD can have a profound impact on patients and their families^3–6^. Early diagnosis of AD is a crucial component in establishing a comprehensive elderly healthcare system, aiming to significantly alleviate the burdens on patients, families, and society^7,8^.

The symptoms of early AD include^1–8^:

- Memory loss: Short-term memory is weakened, while long-term memory is clear. In the severe stage, long-term memory is also lost.
- Spatial orientation disorder: it is easy to get lost in familiar environments.
- Object recognition disorder: Facial recognition ability decreases.
- Decreased cognitive ability: Difficulty in processing complex information and tasks
- Personality and behavior changes: There are large mood swings, irritability, anxiety, and depression.

### Explanation of the symptoms

It is the popular view currently that brain tissue damage involving memory function, such as damage to the hippocampus, is the most common cause of dementia. Brain tissue damage mainly comes from the attack of toxic substances. The toxic substance hypothesis mainly includes the β-amyloid hypothesis and the Tau protein hypothesis^1–8^.

### Wondering about the explanation

- The brain's memory system is a whole, including both the tissues that store information, such as the hippocampus, and the pathways that transmit information, such as the visual information transmission pathway. Any damage to any one of these links will affect memory, not just the information storage organization. The damage to the hippocampus is believed to be more closely related to AD. The hippocampus plays a crucial role in many functions such as memory, learning, spatial navigation, and behavior. However, its primary function is to consolidate and encode memories. Short-term memories are stored in the hippocampus, and if they are repeatedly accessed within a short period, the hippocampus transfers the information to the cerebral cortex where it forms long-term memories^2,9–16^.
- Toxic substances can damage both memory storage tissues and information transmission pathways, as well as any other tissues. Why is the hypothesis of memory storage tissue damage preferred?
- If it is only the memory tissue that is damaged, how to explain the patient's early symptoms: short-term memory loss and long-term memory clarity. The memory tissue should follow the same principle for all information storage. For example, the last author of this paper had a neighbor, he was a typical AD patient. He cannot remember his wife, but he knows the author because his wife has taken care of him for a long time and has no patience with him. However, when the author occasionally helped him open the door and pushed the wheelchair for him, the author was very enthusiastic and patient. He selectively remembers pleasant experiences. If it is the memory storage tissue that is damaged, he should not remember the author if he cannot remember his wife. This indicates that the most severely damaged tissue in early AD patients may not be the memory storage tissue, but the tissue associated with memory storage, such as the visual information transmission pathway. Make an analogy as follows: the first part of the computer to be damaged may not be the storage, but the USB (universal serial bus) interface.

### Introduction of visual information transmission pathway

Visual information accounts for about 80% in all of brain information. So, according to the above discussion, the visual information transmission pathway should be noted, which transmits visual information to the memory storage hippocampus. It was introduced below.

The transmission pathway of visual information is named as Visual–Hippocampal pathway (V–H pathway) in this paper, it transmits information obtained from the retina to the hippocampus.

**Figure 1:**
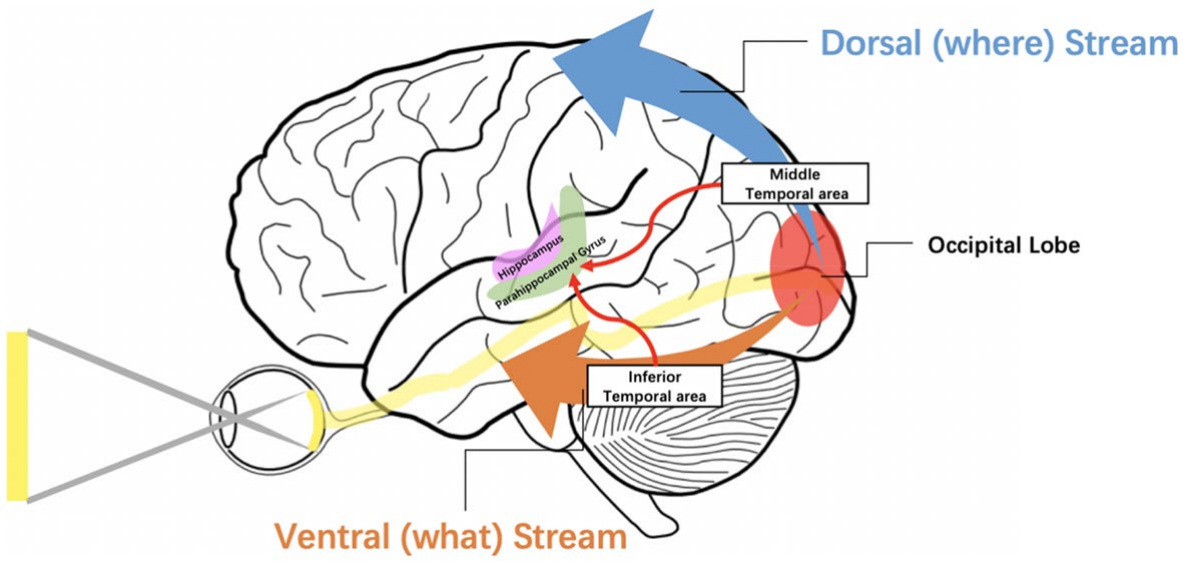
The illustration of the Visual–Hippocampal pathway. Information received by the eyes is processed through two visual pathways after reaching the occipital lobe. The blue portion represents the dorsal visual pathway passing through the middle temporal region, while the orange portion represents the ventral visual pathway passing through the inferior temporal region. After visual processing, the majority of information is transmitted to the parahippocampal gyrus and further relayed to the hippocampus.

V–H pathway includes the five brain tissue regions (Fig. 1): Occipital Visual Cortex (OVC), Middle Temporal Gyrus (MTG), Inferior Temporal Gyrus (ITG), Parahippocampal Gyrus (PHG), and Hippocampus (HC).

OVC, also known as the occipital lobe, is the primary region of the brain responsible for processing visual information. It is located at the back of the brain. It receives signals from the retina and processes them into a representation of the visual scene. OVC is responsible for basic visual functions such as object recognition, motion perception, and spatial attention. OVC is also involved in higher-level cognitive functions such as spatial reasoning, reading, and face recognition^17–20^.

MTG is located in the temporal lobe. It involves in several cognitive functions, including visual-spatial orientation recognition, attention, and language comprehension. MTG processes visual information about motion, objects, and faces, helping us to recognize and identify them. MTG is particularly important for processing complex visual stimuli, such as spatial orientation, recognizing objects in natural scenes, or reading facial expressions. Damage to MTG can lead to various cognitive deficits, including visual processing problems, and attention deficits. These deficits can have a significant impact on daily life, affecting spatial orientation recognition, communication, and independent living^21–23^.

ITG is located in the temporal lobe, inferior to the superior temporal gyrus and anterior to the fusiform gyrus. It plays a crucial role in visual processing, especially the recognition and identification of objects. It is responsible for the recognition of complex visual patterns, including faces, objects, and scenes. The ITG also integrates visual information with other sensory inputs and memories to create a comprehensive understanding of the visual world. ITG is highly connected to other brain regions, allowing it to integrate information from multiple sources. It receives input from OVC, and projects to areas involved in memory, attention, and decision-making. Damage to the ITG can lead to visual recognition deficits, such as prosopagnosia (the inability to recognize faces) or associative visual agnosia (the inability to recognize familiar objects). These deficits can have a profound impact on daily life^24,25^.

PHG is located in the medial temporal lobe, adjacent to the hippocampus. It is involved in various cognitive functions, including memory, spatial navigation, and visual processing. It plays a key role in memory formation and retrieval. It processes information about spatial locations, objects, and events, helping us to form memories and later retrieve them. PHC contributes to spatial navigation. It processes spatial information about our environment, helping us to navigate through complex spaces and find our way around. PHG processes visual information about objects, faces, and scenes, helping us to recognize and identify them. PHG receives input from the visual cortex and integrates it with other sensory inputs and memories to create a comprehensive understanding of the world around us. Damage to PHG can lead to various cognitive deficits, including memory impairments, navigation difficulties, and other visual processing problems^26–28^.

HC is located in the medial temporal lobe. It plays a crucial role in memory formation and spatial navigation. Its main function is to convert short-term memories into long-term ones. HC is essential for spatial navigation. It processes spatial information about our environment and helps us to navigate through complex spaces. It also plays a role in memory formation, allowing us to form memories of specific events and autobiographical experiences. Damage to the hippocampus can lead to memory impairments and spatial navigation difficulties^29–32^.

**Figure 2:**
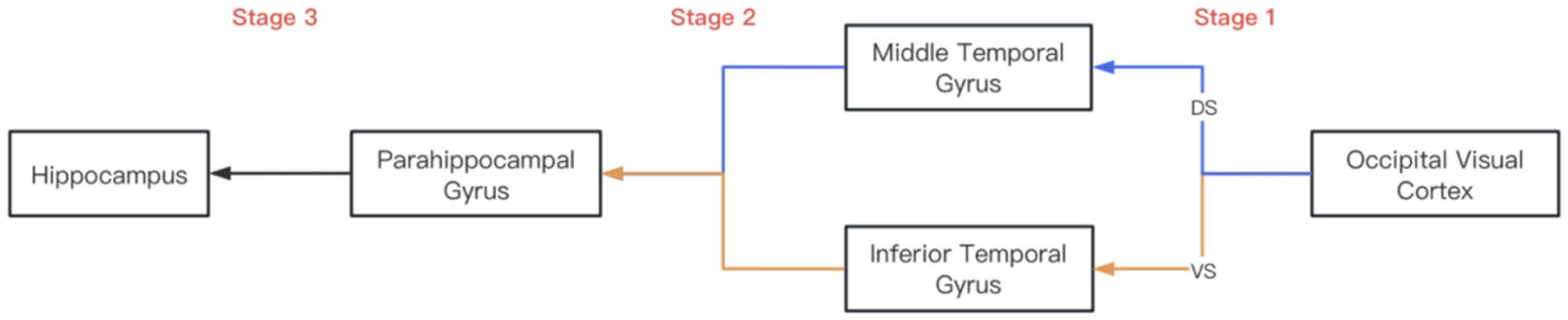
Flowchart of the Visual–Hippocampal pathway. Stage 1 represents the visual processing stage, Stage 2 involves the transmission of visual information to the parahippocampal gyrus, and Stage 3 illustrates the relay from the parahippocampal gyrus to the hippocampus*.*

The above five brain regions of V–H construct two sub-pathways to transmit visual information. One is OVC–MTG–PHG–HC (Fig. 2, top pathway), and the other is OVC–ITG–PHG–HC (Fig. 2, bottom pathway). The first pathway is called the dorsal stream, and the second pathway is called the ventral stream (Fig. 1)^33,34^. These two sub-pathways play distinct roles in analyzing and interpreting visual information. The dorsal stream primarily is associated with motion perception and spatial localization^35^, while the ventral stream involves higher-level visual functions such as object recognition and face identification^36^.

The process of visual information transmission along the V–H pathway is divided into three stages. The first stage is the stage of information acquisition, the second stage is the stage of information transmission, and the third stage is the stage of information saving (Fig. 2). The first stage includes two manners of information acquisition, one is OVC–MTG, the other is OVC–ITG. The first manner is responsible for motion perception and spatial localization, such as spatial orientation. The second manner is responsible for object recognition, such as face recognition. The second stage includes two manners too, one is MTG–PHG, and the other is ITG–PHG. MTG–PHG transmits spatial orientation information, and ITG–PHG transmits object recognition information. The third stage is PHG–HC, which is responsible for saving information in the hippocampus. OVC is the primary visual center, and all visual information comes from it. When the brain performs various complex neural activities, it requires a large amount of ATP to maintain the electrical activity, signal transmission, memory storage and other functions of neurons. Even in the resting state, the brain needs to consume a considerable amount of energy. Therefore, efficient hemoglobin for oxygen transport in the brain is particularly important in maintaining the normal work of brain cells. There is no doubt that a large amount of energy is required in the process of transmitting visual information to the hippocampus for storage. Hemoglobin has become an indispensable link in effectively transporting oxygen. Only by providing enough oxygen can the normal memory processing of visual information be ensured.

### Introduction of haemoglobin

To study the V–H pathway's impact on AD, a research perspective must be chosen. Since the V–H pathway transmits nearly 80% of information, it should cost a lot of energy. Energy supply to the V–H pathway is fundamental. So, energy supply is selected as research perspective. However, it is difficult to detect energy supply of the V–H pathway. Then, hemoglobin expression is selected as a mirror to reflect energy supply, and the reason is as follows. The release of a large amount of energy requires oxygen. And in a certain range, the more oxygen, the more energy is released. Molecular hemoglobin is the carrier of molecular oxygen. The higher the hemoglobin expression, the greater the capacity for carrying oxygen, resulting in a more efficient energy supply on the V–H pathway to support information transmission^37–41^.

Hemoglobin is a crucial protein in the human body, primarily responsible for the exchange and transportation of oxygen and carbon dioxide^42^. The supply of oxygen is vital for normal biological activities in the brain, especially in high-energy-consuming neural tissues^43,44^. Hemoglobin is composed of four subunits: two α subunits (HBA1 and HBA2) and two β subunits (HBB)^45^. Each subunit contains an iron ion that can combine with oxygen to form oxyhemoglobin, allowing oxygen to be transported to the brain tissue regions of the V–H pathway and release energy to transmit information to the hippocampus. In research related to AD, there are also some proofs that hemoglobin related anemia is related to AD^46–49^.

In the hemoglobin molecule, heme combines with its iron atoms to form oxygen. It absorbs oxygen in the lungs and then travels within the tubes to the tissues and organs that need oxygen. The binding and release processes of oxygen are realized through the coordination bonds and dissociation bonds of heme molecules in hemoglobin. However, heme is easily interfered with by other toxic substances, thus affecting the ability of hemoglobin to transport oxygen, such as carbon monoxide, highly phosphorylated tau protein and other toxic substances.

### Research topic of this paper

**Figure 3:**
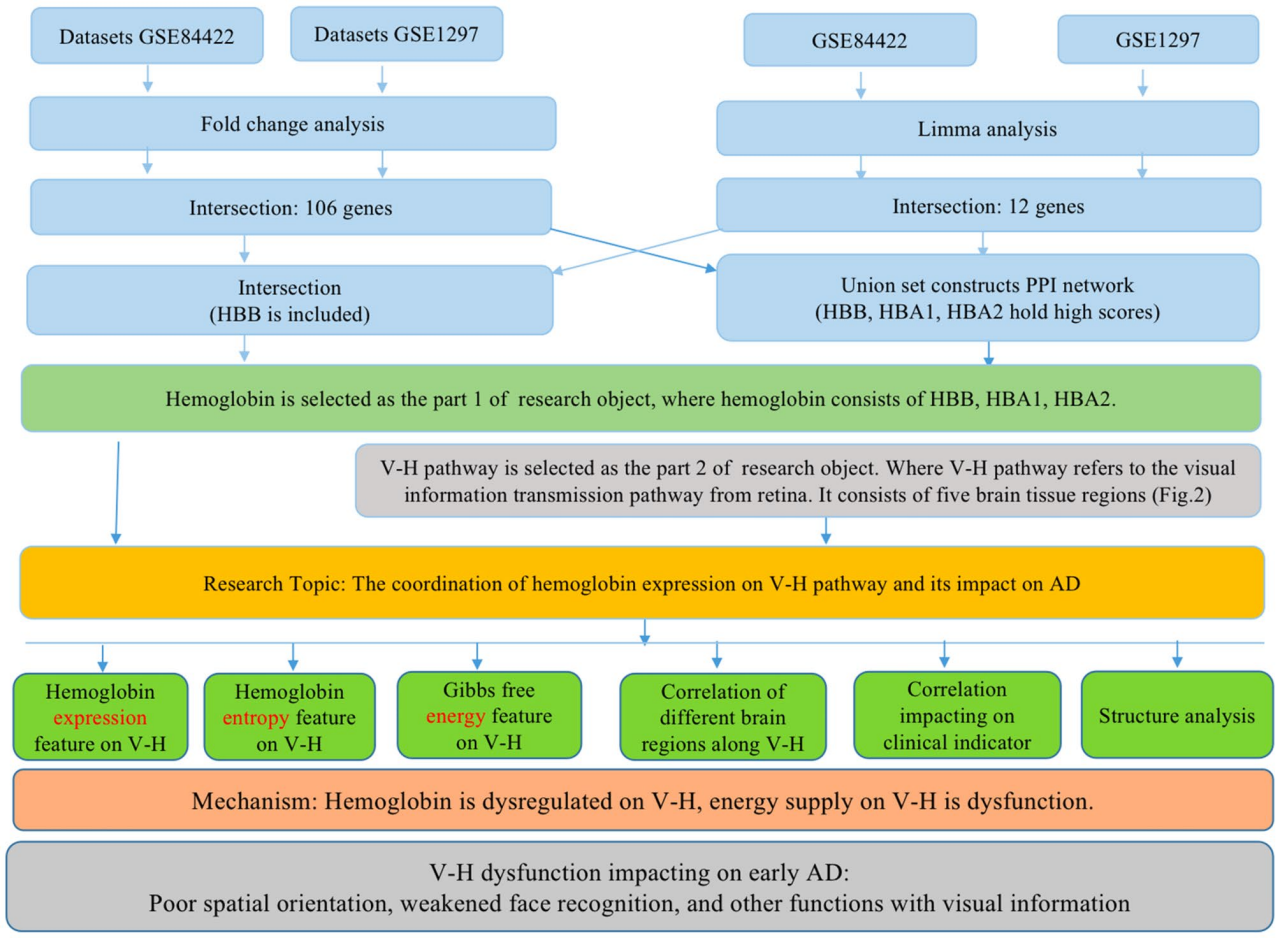
The framework of study.

It is the research topic of this paper that explores the hemoglobin subunit expression features on the V–H pathway to detect the energy supply dysfunction of the V–H pathway. The contents of the study are as follows. And the framework of this study is illustrated in Fig. 3.

- Filter out the genes of hemoglobin subunits from more than 20,000 gene probes.
  The methods are common bioinformatics methods, such as DEGs (differential expression genes) and PPI (protein–protein interaction). All genes filtered out are the candidates of AD genes.
- Drill out the features of hemoglobin subunits on the V–H pathway.
  **Method**
  This paper includes both the methods the authors have used in the past year^46–55^ and the new methods proposed in this paper. The new methods include the calculation of the correlation between different brain regions along the V–H pathway. And also includes the calculation of the entropy of the hemoglobin system. The new methods include the calculation to detect the pathway dysfunction impact on clinical indicators of AD. The new methods include the analysis of Gibbs free energy. In addition, the structure of hemoglobin and its pathogenic mechanism were analyzed.

#### Features

The authors of this paper try to drill out the following features using the above methods: First, hemoglobin expression feature on the pathway. Second, entropy feature on the pathway to detect the disorder of the hemoglobin system. Third, gibbs free energy to detect the oxygen capacity of hemoglobin on the pathway. Fourth, correlation between different brain tissue regions of the V–H pathway to detect the dysfunction of the pathway. Fifth, the dysfunction of the pathway impacts on clinical indicator of AD. Sixth, inference of AD between hemoglobin molecule and tau in biochemical structure.

- Deduce the molecular mechanism that the V–H pathway impacting on AD from the above features.
- Explain the early symptoms of AD using the mechanism.

## Result

### Filter out significantly differential expression genes

#### Method

Apply two methods to filter out the candidates of AD genes that hold significant expression differences.

**Figure 4:**
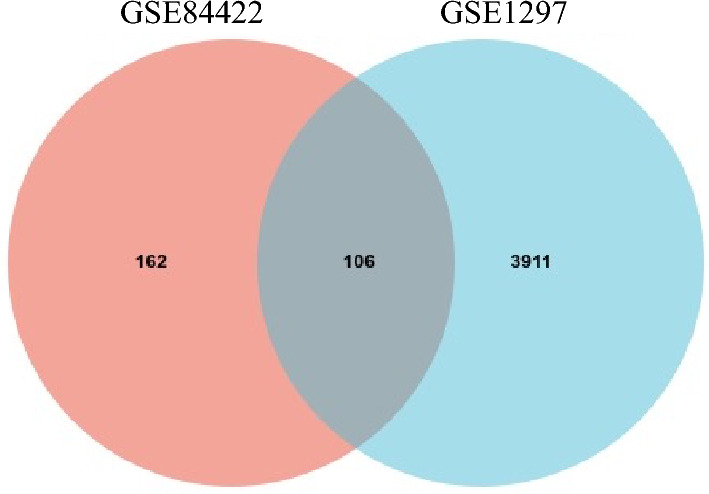
The identification of AD genes using the method of fold change. The genes of hemoglobin subunits are included in the intersection.

**Figure 5:**
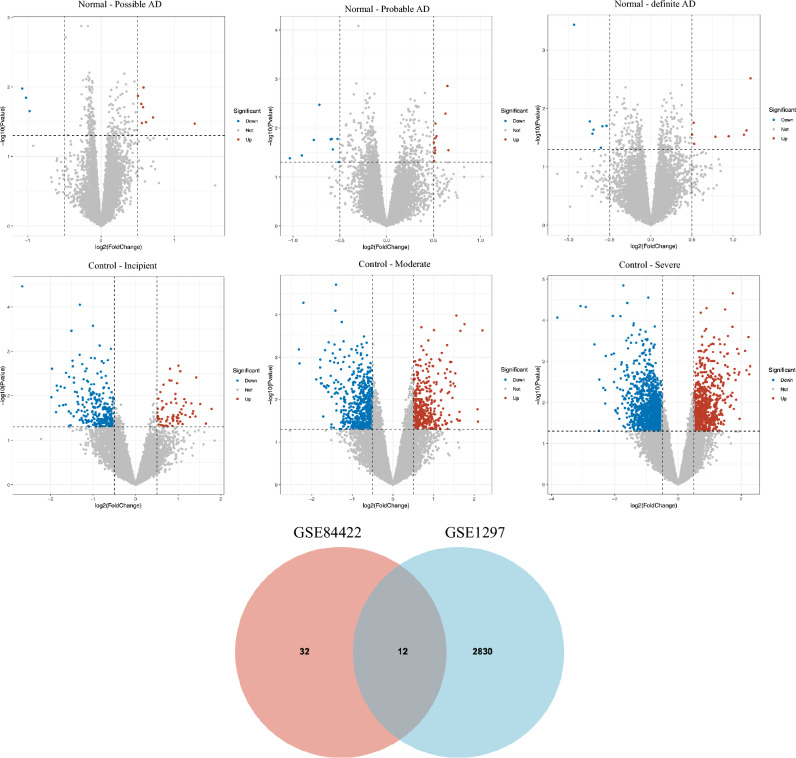
The identification of AD genes using expression difference. The genes of hemoglobin subunits are included in the intersection.

- The method of fold change analysis (Fig. 4). The genes whose expression levels are higher or lower than average expression levels significantly are selected, where the criteria of significance are that Fold_Change ≥ 1.5. The method acts on two independent datasets GSE84422 and GSE1297 respectively, candidate genes are selected into two sets, then their intersection is the output.
- The method of Volcano plot (Fig. 5). For every gene, the difference between two states AD and control is used as the input of the Limma differential expression method, then the genes with a significant difference are selected, where the criteria of significance were set as P-value ≤ 0.05, |log_2_FC|≥ 0.5. The method acts on two independent datasets GSE84422 and GSE1297 respectively, candidate genes are selected into two sets, then their intersection is the output.

#### Input data

All data were from GSE84422 and GSE1297 datasets, NCBI website. GSE84422 includes four states, Normal, Possible AD, Probable AD, and Definite AD. GSE1297 includes four stages of AD, Control, Incipient, Moderate, and Severe. All raw data were logarithmized and normalized, and their exponents were used for size comparison. In this way, only the magnitude of each number was compared, minimizing the impact of noise as much as possible.

#### Output data

Figures 4 and 5 illustrate the output.

#### The special feature of output

Using two independent datasets and two different methods, still, the hemoglobin subunits are filtered out. That is, hemoglobin subunits hold significant abnormal expression as AD progression, it is very possible that they are related to AD.

### Hemoglobin subunits are key nodes in the PPI network

#### Method

Construct a protein–protein interaction network (PPI network) using the STRING website (version11.string-db.org).

#### Input data

The 106 genes from the fold change analysis (Fig. 4) and the 12 genes identified from the Limma analysis (Fig. 5) were grouped and subjected to the PPI network.

#### Output data

**Figure 6:**
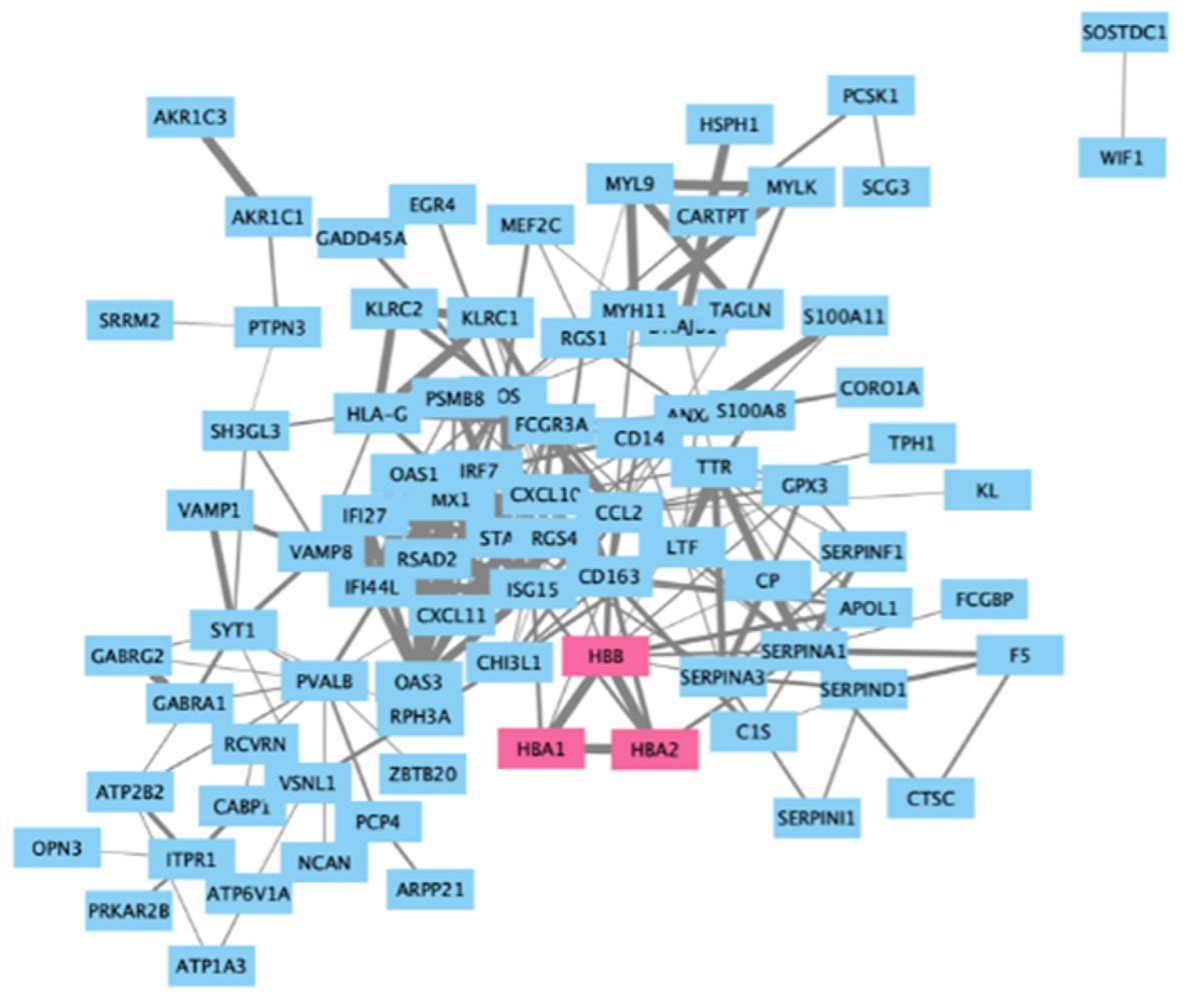
PPI Network. Hemoglobin subunits form a minimized closed loop.

Figure 6 is the output of the PPI network.

#### The special feature of output

The hemoglobin subunits HBB, HBA1 and HBA2 hold high confidence associated with AD (The thicker the line, the more confident it is.). Hemoglobin subunits form a closed loop in PPI, demonstrating a marked correlation between these three genes.

### The expression feature of hemoglobin subunits on the visual information transmission pathway

#### Method

List the expression levels of hemoglobin subunits on the pathway that transmits visual information (V–H pathway), and observe the feature of change as AD progresses.

#### Input data

The different brain regions of the V–H pathway and the gene expression levels in these regions. The brain regions consist of the Occipital visual cortex, Middle temporal gyrus, Inferior temporal gyrus, Parahippocampal gyrus, and Hippocampus. The original data are from GSE84422.

#### Output (Fig. 7)

**Figure 7:**
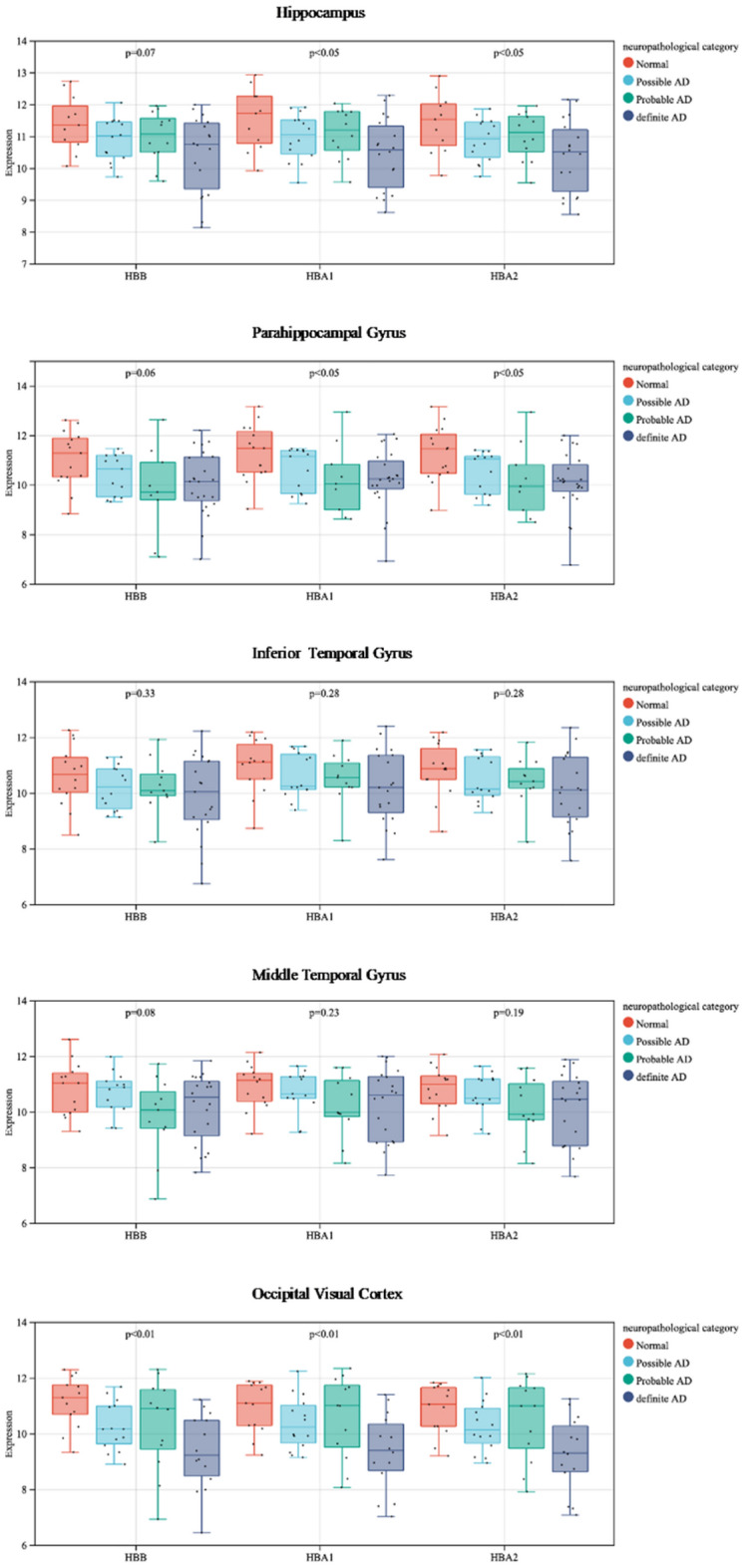
Expression levels of hemoglobin subunits on V–H Pathway. The expression levels exhibited a decreasing trend with the onset of AD.

The expression levels of hemoglobin subunits in different brain regions are visualized and displayed using box-and-whisker plots, based on mean expression values.

#### The special feature of the output

- On the brain regions of the V–H pathway, the hemoglobin subunits are down-regulated overall with AD progression.
- On the regions of the Inferior temporal gyrus and Hippocampus, the subunits are consistently down-regulated with AD progression.
- Guess: The down-regulation feature suggests that, as AD progresses, on the pathway of visual information transmitted, the number of hemoglobin decreases gradually overall, the oxygen supply becomes weaker, the energy supply becomes weaker, and then dysfunction on the V–H pathway happens. That is, dementia not only comes from memory loss of the hippocampus but also from damage to the information transmission pathway. The following work of this paper is all based on the argumentation of this hypothesis.

### Hemoglobin system becomes disordered at early stage of AD

#### Method

The hemoglobin system consists of subunits HBB, HBA1, and HBA2. Under the disturbing of toxic substances, their expression becomes disorder possibly, and the coordination of the three subunits becomes weak. That is, the entropy of the hemoglobin system will change as AD progresses and toxic substances accumulate.

In this section, the entropy of the hemoglobin system was calculated. AD progression passes through four stages, Normal, Possible AD, Probable AD, and Definite AD. So, there are four values of entropy. The trend of entropy changing reflects the disorder of the system with AD progression.

V–H pathway includes five brain tissue regions. At each region, the entropy was calculated in this section. If the entropy at some region becomes significantly high and more disordered, then the pathway has the risk of dysfunction.

#### Input data

The original data are from GSE84422. The expression levels of the subunits of hemoglobin HBB, HBA1, and HBA2, are sampled from the brain tissue regions of the V–H pathway. The entropy of every region is calculated, which measures the disorder of the region. The entropies at the four stages are calculated, which are Normal, Possible AD, Probable AD, and Definite AD respectively.

#### Output

**Figure 8:**
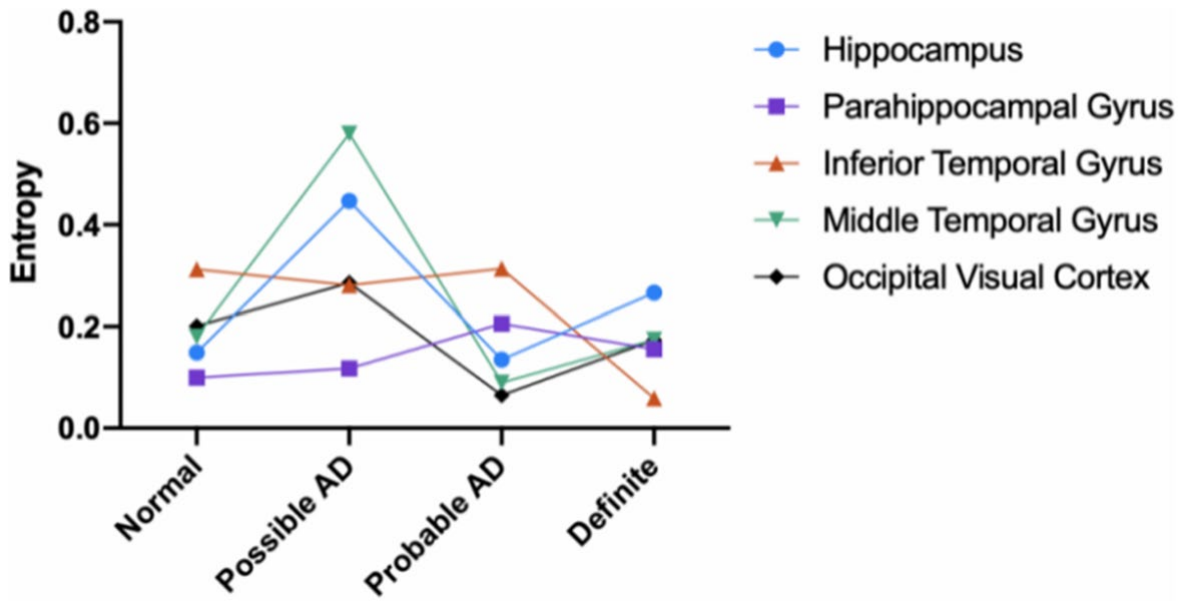
The trend of entropy of the hemoglobin system in the V–H pathway. At early AD stage, brain regions MTG and HC hold increased entropy and hemoglobin expression becomes more disordered.

For each brain tissue region, the trend of entropy with AD progression is described visually by curves and presented in Fig. 8.

#### The special feature of the output

- At the stage of Possible AD, the entropy of most brain regions markedly increased.
- In the Middle Temporal Gyrus (MTG) and Hippocampus (HC) regions, entropy increase is significant at early stage of Possible AD.
- At early stage, the hemoglobin system becomes disordered, and the oxygen supply on the V–H pathway becomes inadequate.
- MTG is responsible for spatial orientation, so the disorder leads to orientation disorder.
- The entropy in HC becomes high, the disorder of HC increases, V–H pathway holds a high risk of dysfunction at early stage. So, at early stage, the patient holds a high risk of spatial orientation disorder and object recognition disorder.

### Gibbs free energy analysis

#### Preliminary: Gibbs free energy

##### Gibbs free energy, G

Gibbs free energy expresses the amount of energy capable of doing work during a reaction at constant temperature and pressure. When a reaction proceeds with the release of free energy (that is when the system changes to possess less free energy), the free-energy change, ΔG, has a negative value and the reaction is said to be exergonic. In endergonic reactions, the system gains free energy and ΔG is positive^56^.

##### Enthalpy, H

Enthalpy is the heat content of the reacting system. It reflects the number and kinds of chemical bonds in the reactants and products. When a chemical reaction releases heat, it is said to be exothermic; the heat content of the products is less than that of the reactants, and the change in enthalpy, ΔH, has, by convention, a negative value. Reacting systems that take up heat from their surroundings are endothermic and have positive values of ΔH^56^.

##### Entropy, S

Entropy is a quantitative expression of the randomness or disorder in a system. When the products of a reaction are less complex and more disordered than the reactants, the reaction is said to proceed with a gain in entropy^56^.

##### The relationship between energy and entropy

Under the conditions existing in biological systems (including constant temperature and pressure), changes in free energy, enthalpy, and entropy are related to each other quantitatively by the equation^56^.

1 $$\Delta G=\Delta H-T \Delta S,$$

in which ΔG is the change in Gibbs free energy of the reacting system, ΔH is the change in enthalpy of the system, T is the absolute temperature, and ΔS is the change in entropy of the system.

Living organisms preserve their internal order by taking from the surroundings free energy in the form of nutrients or sunlight, and returning to their surroundings an equal amount of energy as heat and entropy^56^.

### The energy supply of hemoglobin becomes weaker at early stage of AD

According to Fig. 7, it is known that, at early stage, and at most of brain tissue regions of the V–H pathway, the expressions of hemoglobin subunits are disordered. The disorder interferes with the biochemical processes in which hemoglobin is involved, affecting oxygen transport. This disorder will increase the entropy ΔS of the reacting system in which hemoglobin is involved.

Under ideal conditions, such as constant temperature and pressure, enthalpy ΔH is constant, because enthalpy refers to the energy saved in the chemical bond and the energy of system to do work on the outside which is a constant under the ideal condition.

So according to formula (1), we have the following differential expression

2 $$d\left(\Delta G\right)=-T d(\Delta S)$$

To understand formula (2), we give the following example: If the entropy increases by 0.02 units (i.e., $d\left(\Delta S\right)=0.02$), the free energy will decrease by 0.02T (i.e., $d\left(\Delta G\right)=-0.02T$). That is to say, the energy involved in the biochemical reactions of hemoglobin will decrease by 0.02T, which will reduce its oxygen transport capacity.

Therefore, the following conclusions can be drawn: During early stage of AD, at the brain tissue regions of the V–H pathway, the hemoglobin system holds increased entropy, and then the system becomes more disordered. This disorder dissipates energy saved in hemoglobin, then the capacity of hemoglobin to transport oxygen becomes weak, the energy supply of the V–H pathway becomes weak, and at last, the dysfunction risk of the V–H pathway increases. Ultimately, patient holds a high risk of spatial orientation disorder and object recognition disorder at early stage.

### The correlation between different brain regions becomes weaker at early stage of AD

#### Method

Calculate the Pearson correlation coefficients between different brain tissue regions of the V–H pathway.For example, along the V–H pathway, MTG is neighbor to PHG (Fig. 2), and MTG–PHG is one of a section of the pathway. The hemoglobin expression at the two neighboring brain regions should be correlated if the patient is at normal state. The correlation between the two neighboring regions was calculated.

When the expression levels of three subunits of hemoglobin form a 3-dimensional vector on MTG, it was denoted as

3 $$\overrightarrow{{x}_{MTG}}=\left(HBB, HBA1, HBA2\right)$$

The vector $\overrightarrow{{x}_{MTG}}$ is called as the hemoglobin expression on brain region MTG.

As a same, hemoglobin expression at region PHG is denoted by $\overrightarrow{{x}_{PHG}}$.

The correlation between the two vectors is defined as the Pearson correlation coefficient of two unit vectors:

4 $${R}_{MTG-PHG}=\frac{\sum (\overrightarrow{{x}_{i}}-\overline{x })(\overrightarrow{{y}_{i}}-\overline{y })}{\sqrt{\sum {\left(\overrightarrow{{x}_{i}}-\overline{x }\right)}^{2}\sum {\left({\overrightarrow{{y}_{i}}}-\overline{y }\right)}^{2}}},$$

where $\overrightarrow{{x}_{i}}$ presents a 3-dimensional vector from a sample in MTG, and ${y}_{i}$ presents vector from PHG.

If the value ${R}_{MTG-PHG}$ is close to zero at AD stage, the correlation of the two regions is meager, it is not coordinated that the hemoglobin expression on the two regions. That is, the oxygen supply in the two neighboring regions is not coordinated, and this coordination will lead to the dysfunction of visual information transmission.

#### Input data

The raw data are from dataset GSE84422. The input data is logarithmically transformed and only the exponential is compared, which eliminates noise pollution. Then all data are standardized, making data from different brain regions and different samples comparable.

#### Output

**Figure 9:**
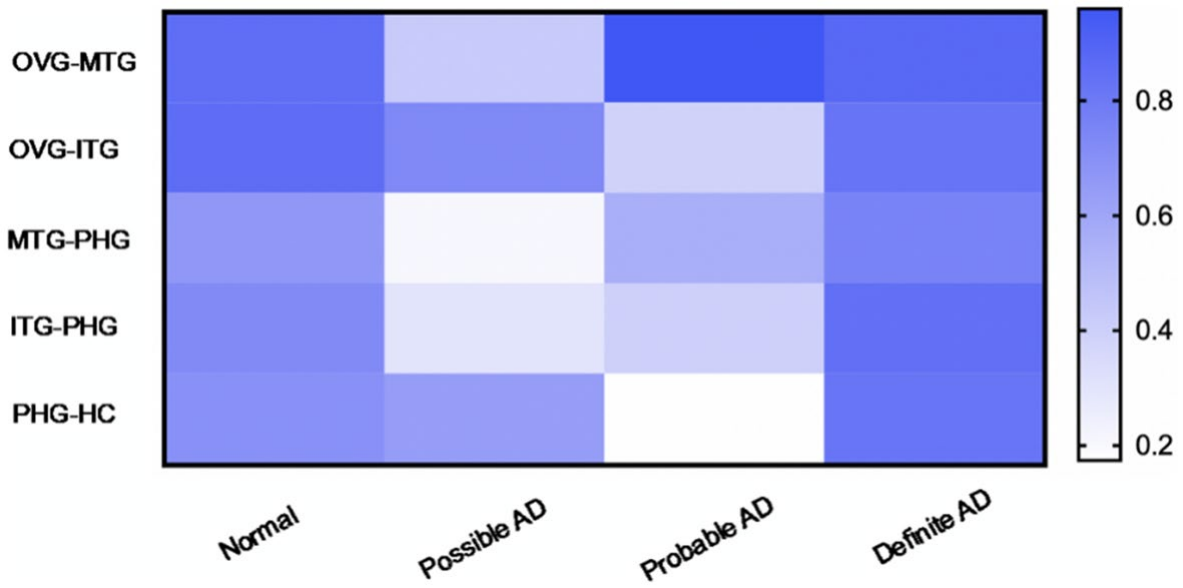
The correlation between different brain regions along the V–H pathway. The correlation MTG–PHG becomes weak significantly, the information transmission becomes weak, where the information is responsible for spatial orientation. So, the earliest symptom is spatial orientation disorder, and the symptom is high risk. At early stage, PHG–HC becomes weak significantly, and saving information becomes weak. So, the two symptoms are high-risk spatial orientation disorder and object recognition disorder.

The correlations between different regions are listed in Fig. 9.

#### The special feature of the output

- At the earliest stage (Possible AD), the correlation between MTG and PHG is close to zero (i.e., ${R}_{MTG-PHG}\approx 0$). Section MTG–PHG of the V–H pathway is responsible for transmitting the information of spatial orientation. So it is a high risk that the patient holds weak spatial orientation at the earliest stage.
- At early stage (Probable AD), the correlation between PHG and HC is close to zero. Section PHG–HC is responsible for saving information in the hippocampus, and the information includes spatial orientation and object recognition. So, it is a high risk that the patient holds weak spatial orientation and face recognition at early stage.
- At early stage (Possible AD and Probable AD), the correlation between brain regions becomes weak, and the dysfunction of the V–H pathway is possible. So, it is a high risk that the patient holds weak spatial orientation and face recognition.

### The discoordination of the V–H pathway is related to neurofibrillary tangles

#### Conceptions to measure correlation between two brain regions

Hemoglobin consists of three subunits HBB, HBA1, HBA2.

Hemoglobin expression is defined as the vector (HBB, HBA1, HBA2), where the expression level of the three subunits is abstracted as the three components of the vector respectively.

If the vector is measured at brain region OVC, it is denoted by $\overrightarrow{{x}_{OVG}}$. V–H pathway consists of five brain regions, OVC, MTG, ITG, PHG, HC. So, for a sample, there five vectors, $\overrightarrow{{x}_{OVG}}$, $\overrightarrow{{x}_{MTG}}$, $\overrightarrow{{x}_{ITG}}$, $\overrightarrow{{x}_{PHG}}$, $\overrightarrow{{x}_{HC}}$.

For *n* samples, there are *5n* vectors, every region has *n* vectors.

The size of Hemoglobin expression is defined as the length of vector, such as $| \overrightarrow{{x}_{OVG}}|$. The length $| \overrightarrow{{x}_{OVG}}|$ reflects the total expression of the three subunits on region OVC. If region OVC contains many molecular hemoglobin, the hemoglobin expression on the region is high, the three subunits hold high expression also, then the size of hemoglobin on OVC is big (i.e., $| \overrightarrow{{x}_{OVG}}|$ is big).

Size correlation between two brain regions is defined as the product of two sizes of hemoglobin expressions. For example, $\left|\overrightarrow{{x}_{OVG}}\right|\cdot |\overrightarrow{{x}_{MTG}}|$ is the size correlation between region OVC and MTG. The bigger the size correlation, the richer hemoglobin in these two neighbor regions in general. At least, it is not possible that any of the two regions contains very little molecular hemoglobin. Size correlation reflects the coordination of hemoglobin supply on the two neighbor regions of the V–H pathway. If the size correlation becomes very small as AD progresses, the coordination of oxygen supply on the V–H pathway is impaired.

Proportion correlation between two brain regions is defined as the cosine of the angle between two vectors of the two regions. For example, for the two neighbor regions MTG and PHG, there are two vectors $\overrightarrow{{x}_{MTG}}$ and $\overrightarrow{{x}_{\text{PHG}}}$. And the proportion correlation is ${R}_{MTG-PHG}=cos\theta$, where $\theta$ denotes the angle between the two vectors. i.e.,

$${R}_{MTG-PHG}=\frac{\overrightarrow{{x}_{MTG}}}{|\overrightarrow{{x}_{MTG}}|}.\frac{\overrightarrow{{x}_{\text{PHG}}}}{|\overrightarrow{{x}_{\text{PHG}}}|}$$

Every molecular hemoglobin contains two HBB, one HBA1, and one HBA2, so the proportion is HBB: HBA1: HBA2 = 2:1:1. In an ideal model, every region holds the ideal proportion 2:1:1. So, the vector of every region is proportional to vector (2, 1, 1). Then, if any proportion correlation is $cos\theta =1$ the correlation is perfect. However, under the impact of toxic substances, the standard proportion 2:1:1 is damaged. For example, in the MTG region, the hemoglobin expression is proportional to vector (0.3, 0.2, 0.3). That is, toxic substances make HBB expression decrease severely (from 2 units to 0.3 units. On the PHG region, the hemoglobin expression is proportional to vector (0.2, 0.4, 0.4). Thus $cos\theta =0.26$, suggests that it is not coordinated hemoglobin expressions on the neighbor regions of the V–H pathway, the oxygen supply on the two regions is not coordinated, and the dysfunction of energy supply on the two regions holds high risk.

Synthesized correlation between two regions is defined as the inner product of between two vectors of two regions. For example, for the two neighbor regions MTG and PHG, there are two vectors $\overrightarrow{{x}_{MTG}}$ and $\overrightarrow{{x}_{\text{PHG}}}$. The inner product is $\overrightarrow{{x}_{MTG} }. \overrightarrow{{x}_{\text{PHG}}}$.

If the inner product becomes very small as AD progresses, either the size of hemoglobin expression on the two neighbor regions is not coordinated, or the proportion of hemoglobin subunits is not coordinated. Any discoordination will impact on V–H pathway and damage the oxygen supply on the pathway, resulting in dysfunction of the energy supply on the pathway. At last, the visual information transmission along the V–H pathway is impaired, and this impairment will lead to spatial orientation disorder and object recognition disorder.

In the following third level sections 2, 3, and 4, it will be analyzed that the relationship between neurofibrillary tangles (NFTs) and the correlation among different brain regions. In “The relationship between NFTs and the size correlation of two neighbor brain regions”, it was discussed that the relationship between NFTs and the size correlation of hemoglobin. In “The relationship between NFTs and the proportion correlation of two neighbor regions”, the relationship between NFTs and the proportion correlation of hemoglobin was discussed. In “The relationship between NFTs and synthesized correlation of neighbor regions”, the relationship between NFTs and the synthesized correlation was discussed.

### The relationship between NFTs and the size correlation of two neighbor brain regions

#### Method

There is the following one-to-one correspondence in the data structure of this paper: For a given sample (or patient), a brain region ~ a NFT value ~ a vector of hemoglobin expression (HBB, HBA1, HBA2).

So, we have that,

Sample 1: region OVC~$m$ (NFT value) ~ $|\overrightarrow{{x}_{OVG}}|$ (size of hemoglobin expression).

Sample 2: region MTG~$n$ (NFT value) ~ $|\overrightarrow{{x}_{MTG}}|$

Then, the size correlation between OVC and MTG is a pair of orders: $(\sqrt{\text{mn}}, \left|\overrightarrow{{x}_{OVG}}\right|\cdot |\overrightarrow{{x}_{MTG}}|$), where $\sqrt{\text{mn}}$ presents the geometric mean. In essence, it is still NFTs.

All of the correlations between two neighbor regions can be calculated as above, and a set of 2-dimensional points can be plotted.

#### Input data

The raw data are from dataset GSE84422. The input data is logarithmically transformed and only the exponential is compared, which eliminates noise pollution. Then all data are standardized, making data from different brain regions and different samples comparable.

#### Output

**Figure 10:**
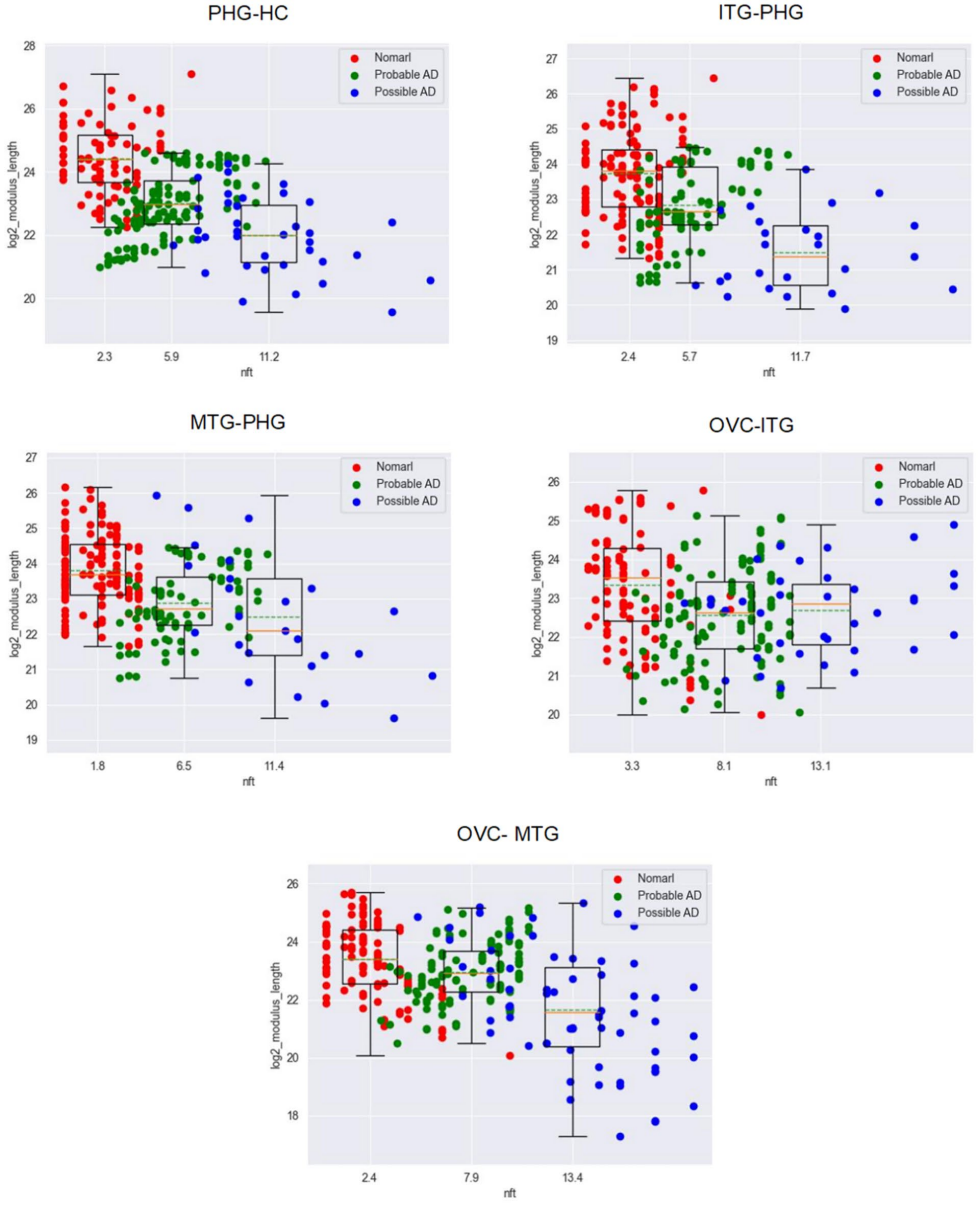
The relationship between NFTs and the size correlation of hemoglobin expression at two neighbor regions.

All points are plotted in Fig. 10.

#### The special feature of output

- The average of all data of size correlations decreases with the increase of NFTs. That is the more dis-coordinated hemoglobin expression on neighbor regions, the bigger the NFTs, the more severe AD, and the weaker spatial orientation recognition and object recognition.
- The variance of size correlation increases with the increase of NFTs. That is, hemoglobin expression loses control gradually on the V–H pathway and becomes more disordered, the more disorder spatial orientation recognition and object recognition.

### The relationship between NFTs and the proportion correlation of two neighbor regions

#### Method

According to Sect. 2, we have a similar example,

Sample 1: region OVC ~ $m$ (NFT value) ~ $\overrightarrow{{x}_{OVG}}$

Sample 2: region MTG ~ $n$ (NFT value) ~ $\overrightarrow{{x}_{MTG}}$

Then, the proportion correlation between OVC and MTG is a pair of orders: $(\sqrt{\text{mn}}, cos\theta$), where

$$cos\theta =\frac{\overrightarrow{{x}_{\text{OVC}}}}{|\overrightarrow{{x}_{\text{OVC}}}|}.\frac{\overrightarrow{{x}_{MTG}}}{|\overrightarrow{{x}_{MTG}}|}.$$

All of the proportion correlation between two neighbor regions can be calculated as above, and a set of 2-dimensional points can be plotted.

#### Input data

See Section 2.

#### Output

Figure 11.

**Figure 11:**
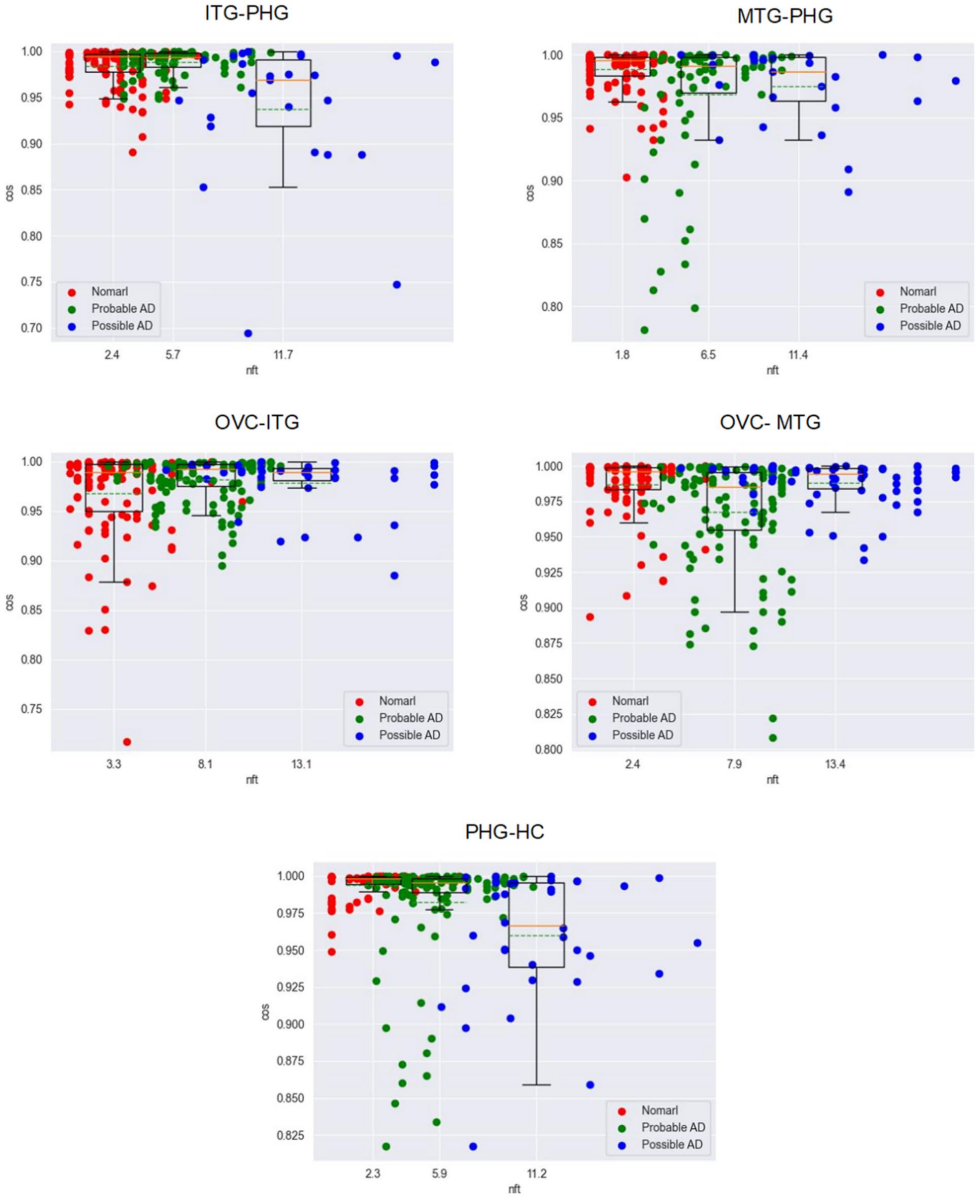
The relationship between NFTs and the proportion correlation of two neighbor regions.

#### The special features of output

As AD occurs, the data distribution becomes more disordered, and the correlation of the proportion of the three subunits becomes disordered. That is, the hemoglobin expression on a region cannot stimulate the response of adjacent brain regions or stimulation becomes weak and disorder. The disorder leads to the dysfunction risk of the V–H pathway, and spatial orientation and object recognition become disorders.

### The relationship between NFTs and synthesized correlation of neighbor regions

#### Method

Calculate the inner product between two vectors of hemoglobin expression at two neighbor brain regions along the V–H pathway. At the same time, the corresponding NFTs value is obtained by multiplying the corresponding NFTs values of the two samples by the square root.

According to Sect. 2, we have similar example,

Sample 1: region OVC ~ $m$ (NFT value) ~ $\overrightarrow{{x}_{OVG}}$

Sample 2: region MTG ~ $n$ (NFT value) ~ $\overrightarrow{{x}_{MTG}}$

Then, the synthesized correlation between OVC and MTG is a pair of orders: $(\sqrt{\text{mn}}, \overrightarrow{{x}_{OVG}}.\overrightarrow{{x}_{MTG}}$), where $\overrightarrow{{x}_{OVG}}.\overrightarrow{{x}_{MTG}}$ presents inner product.

All of the synthesized correlations between two neighbor regions can be calculated as above, and a set of 2-dimensional points can be plotted.

#### Input data

See section 1.

#### Output

Figure 12.

**Figure 12:**
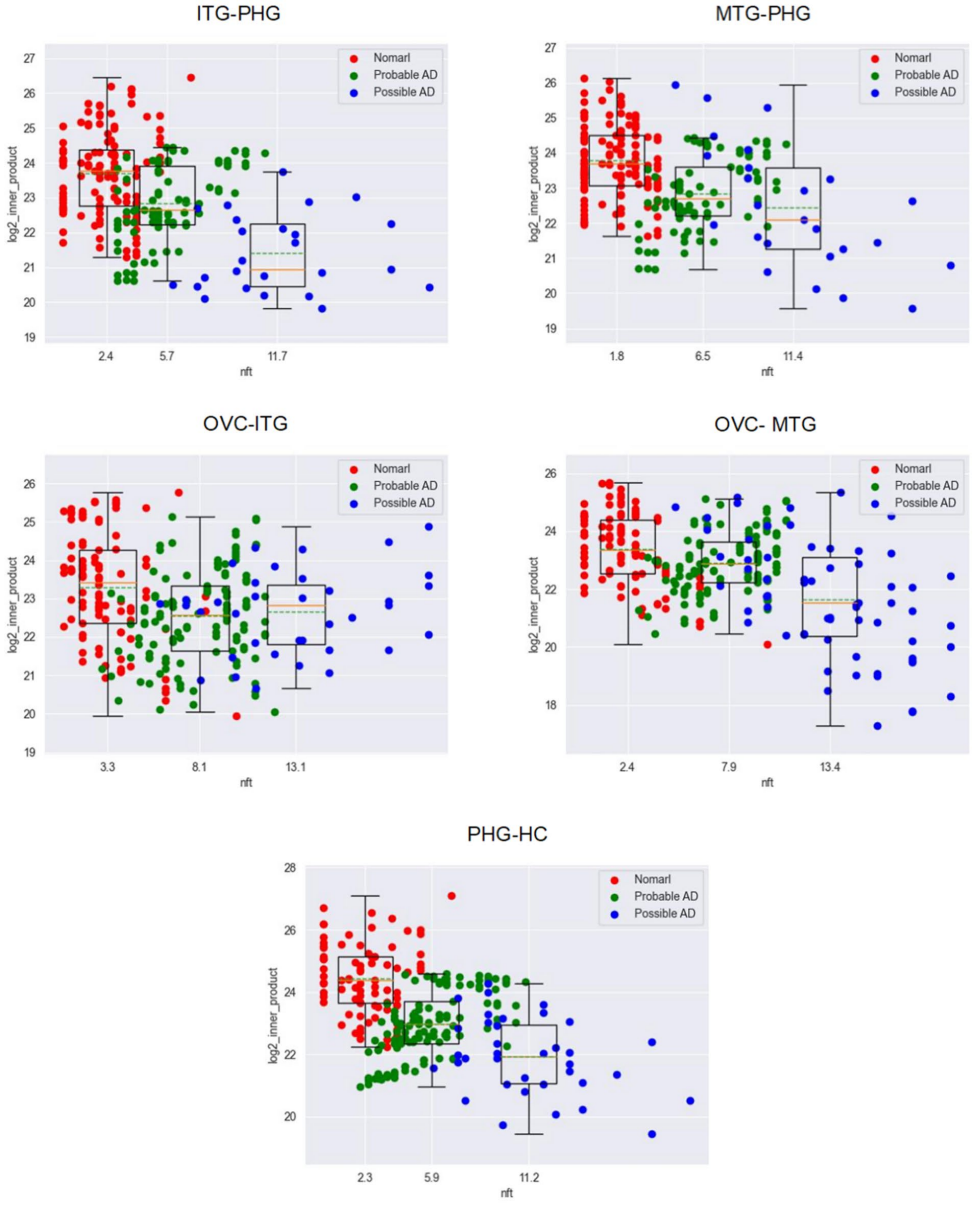
The relationship between NFTs and synthesized correlation of neighbor regions.

#### The features of the output

- As NFTs increase, synthesized correlation decreases significantly.
- As NFTs increase, synthesizedc correlation becomes more disordered significantly.
- The feature of isolation of islands: Hemoglobin expression in one brain region is not synchronized with its expression in neighboring regions, and tends to be independent. So, the coordination of the V–H pathway becomes weak, the oxygen supply becomes disorder, and the energy supply becomes disorder. Then, visual information transmission is impaired. And this impairment impacts spatial orientation recognition and object recognition. At last, the early symptoms appear as spatial orientation recognition disorder and object recognition disorder.

### Hypothesis

#### The features of the V–H pathway

The V–H pathway is the pathway of visual information transmission, which transmits information from the retina to the hippocampus. It consists of five brain tissue regions (Figs. 1, 2): OVC, MTG, ITG, PHG, and HC.

V–H pathway includes two sub-pathways. And one is OVC–MTG–PHG–HC (Fig. 2), named dorsal stream (Fig. 1). Dorsal stream mainly conducts visual information related to spatial perception, target positioning, and motion observation. The other sub-pathway is OVC–ITG–PHG–HC, named ventral stream (Fig. 1). Ventral stream is mainly related to the processing of visual information such as recognition, shape, and color of visual stimuli.

Visual information transmission goes through three stages (Fig. 2): obtaining and organizing information (Stage 1), transmitting information (Stage 2), and storing information (Stage 3). Section OVC–MTG and OVC–ITG belong to the first stage, MTG–PHG and ITG–PHG belong to the second stage, and PHG–HC is the third stage.

To transmit visual information, along the V–H pathway, a brain region should coordinate with its neighboring region. Especially, it is necessary that the energy supply of different brain regions should be coordinated. The release of most energy is determined by the oxygen supply. To release more energy, more oxygen is needed. The oxygen supply is determined by the expression level of hemoglobin. More hemoglobin expression determines more oxygen supply. Therefore, the hemoglobin expression in each brain region reflects the state of energy supply of each brain region like a mirror. More importantly, studying the correlation between hemoglobin expression in various brain regions can detect the coordination of the energy supply of the V–H pathway.

Molecular hemoglobin consists of three subunits, HBB, HBA1, and HBA2. So, the expression levels of the three subunits form a vector (HBB, HBA1, HBA2), and this vector is the hemoglobin expression. For a given sample (patient) and a given brain, the expression levels of the three subunits can be measured, and then the hemoglobin expression is known. Studying the correlation among the vectors of hemoglobin expression will detect the coordination of different brain regions along the V–H pathway.

After studying the correlation among brain regions along the V–H pathway, the following features were observed:

- Hemoglobin expression is significantly abnormal as AD occurs (Figs. 4, 5 and 6).
  This feature suggests that hemoglobin is related to AD very possibly.
- Expression feature: Hemoglobin expression in different brain regions is down-regulated gradually as AD progresses overall (Fig. 7).
  This feature suggests that there is not enough molecular hemoglobin to transport oxygen along the V–H pathway and not enough energy to transport visual information.
- Entropy feature: At the earliest stage of AD, possibly AD, the entropy of the hemoglobin system on brain region MTG and HC increases by more than twice (Fig. 8).
  This feature suggests that significant disorder happens on the V–H pathway. The disorder affects oxygen transportation and energy supply and, at last, affects the transmission of visual information. Especially, the degree of disorder in the MTG region increases by nearly threefold, with spatial orientation disorder symptoms posing a high risk at the earliest stage of AD.
- Gibbs free energy feature: The Gibbs free feature of the hemoglobin system decreases.
- This feature suggests that entropy increases and significant disorder dissipates energy, and reduces the ordered energy to transport oxygen molecules.
- The feature of correlation among brain regions: At early stage of AD (possibly AD or probably AD), the correlation between different brain regions becomes weak, especially for MTG–PHG and PHG–HC (Fig. 9).
  This suggests that the coordination of the V–H pathway becomes weak at early stage. The section MTG–PHG of the pathway holds a very weak correlation, declining more than twice. And this suggests that there is a high risk of spatial orientation disorder at the earliest stage of AD. MTG–PHG holds a very weak correlation too, declining more than twice. And this suggests that there is a high risk of spatial orientation disorder, object recognition disorder, and motion barrier at early stage.
- The relationship between the dis-coordination of the V–H pathway and NFTs: As NFTs increases, the V–H pathway loses coordination gradually (Figs. 10, 11, 12), every brain region of the pathway loses correlation with its neighbor region gradually and becomes independent. This feature is called the isolation of islands in this paper. That is, every brain region loses or weakens connections with its neighbor region, resulting in each region acting like an isolated island.
  This suggests that the dis-coordination of the V–H pathway damages visual information transmission obtained from the retina. Then, the early symptoms appear–spatial orientation disorder and face recognition disorder.

#### The structure features of tau protein and NFTs

##### The features of tau protein

**Figure 13:**
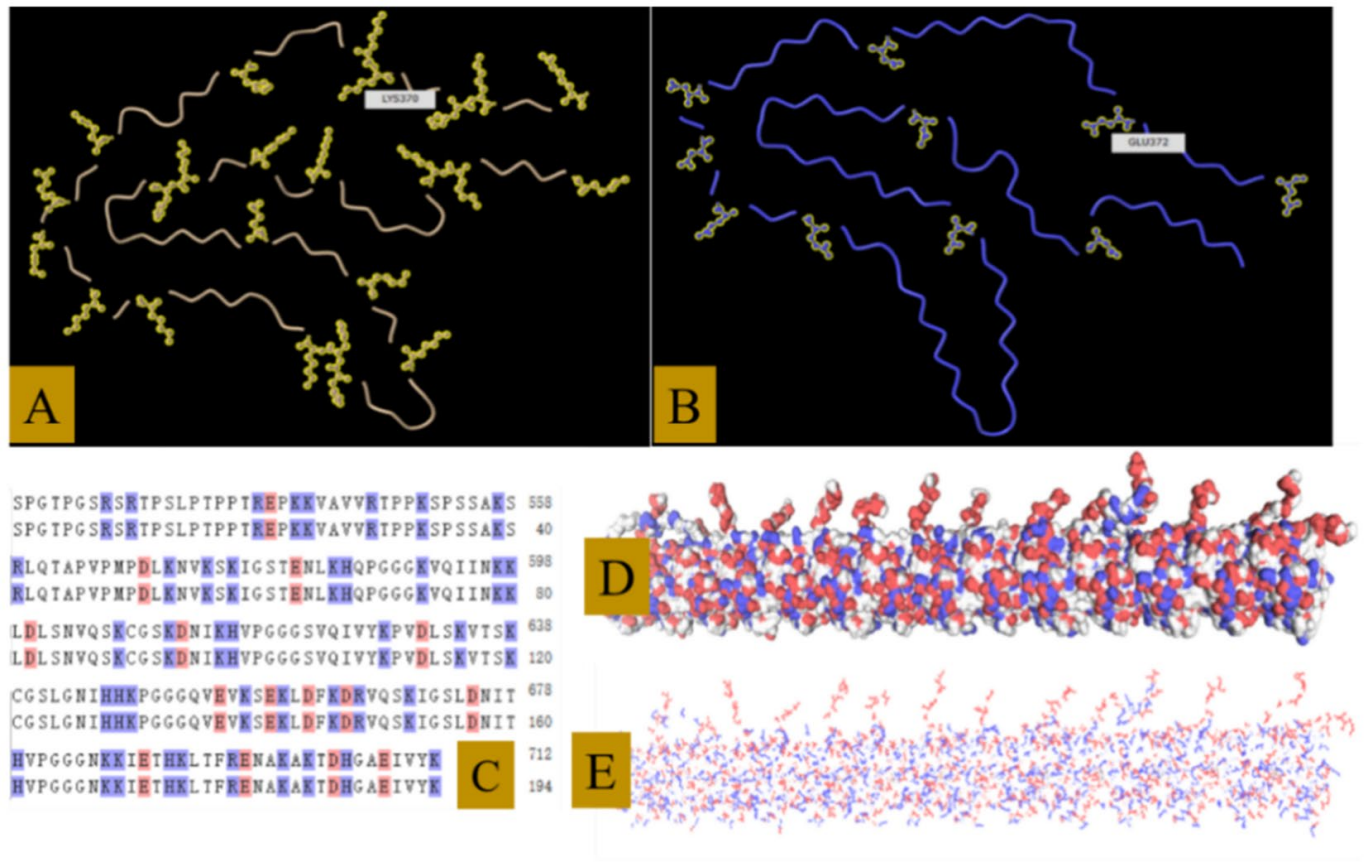
The structure of tau protein (**A**) Distribution of alkali amino acid in a single tau. (**B**) Distribution of acidic amino acid in a single tau. (**C**) The amino acids in a single tau. (**D**) Tau proteins in tubulin. (**E**) Charge distribution (red: negative, blue: positive) (Note: Subfigures (**A**) and (**B**) are generated from website: https://www.ncbi.nlm.nih.gov/Structure/icn3d/full.html?&mmdbid=183918&bu=1&showanno=1&source=full-feature Subfigures (**C**–**E**) are generated from website: https://swissmodel.expasy.org/repository/uniprot/P10636).

- It is a small molecule with highly hydrophilic. Abnormal tau can easily permeate into the bloodstream and bind to hemoglobin. (Fig. 13A–C).
- It has no fixed conformation, and residues exposed (Fig. 13A,B).
- Enrichment of lysine and arginine, which are easily to combine with ions in heme (Fig. 13A).
- A significant quantity of tau is needed to maintain microtubules. And their residues are exposed, resulting in strong activity. Then, there is a high risk of generating abnormal tau (Fig. 13D,E).
- Its residues are easily bound to ions in heme, disrupting the oxygen transport function of hemoglobin (Fig. 13A,B).
- Its mechanism of toxicity is similar to carbon monoxide (CO). Both involve the binding of iron in the heme of hemoglobin to its residues, preventing oxygen from binding to the iron (Fig. 13A,B).
- The toxicity of abnormal tau is stronger than CO because tau contains more residues than the CO molecule (Fig. 13A,B).

##### The features of NFTs

The formation of NFTs is due to the aggregation of proteins within neurons. Specifically, NFTs are formed by the abnormal aggregation of a protein tau. These abnormally aggregated tau proteins form irregular fiber structures and form tangled clumps within neurons. These clumps ultimately lead to the loss of normal function and the eventual death of neurons.

#### The structure features of hemoglobin

##### The features of iron

The electron configuration of an iron atom (Fe) in its outer shell is 3d^6^4s^2^. The two electrons on the outermost layer (i.e., in the 4 s orbital) can be easily lost, resulting in a + 2 valence (Fe^2+^). However, the six electrons on the next outer layer (i.e., in the 3d orbitals) are in an unstable unfilled state, making it susceptible for one additional electron to be lost from the 3d orbitals, resulting in + 3 valence (Fe^3+^). Fe^3+^ is a stable structure with half-filling, so it is more stable than Fe^2+^. The conversion between Fe^2+^ and Fe^3+^ enables hemoglobin to both bind and release oxygen molecules, thus fulfilling its role in oxygen transport, thus achieving the purpose of transporting oxygen by hemoglobin.

##### The features of Heme

**Figure 14:**
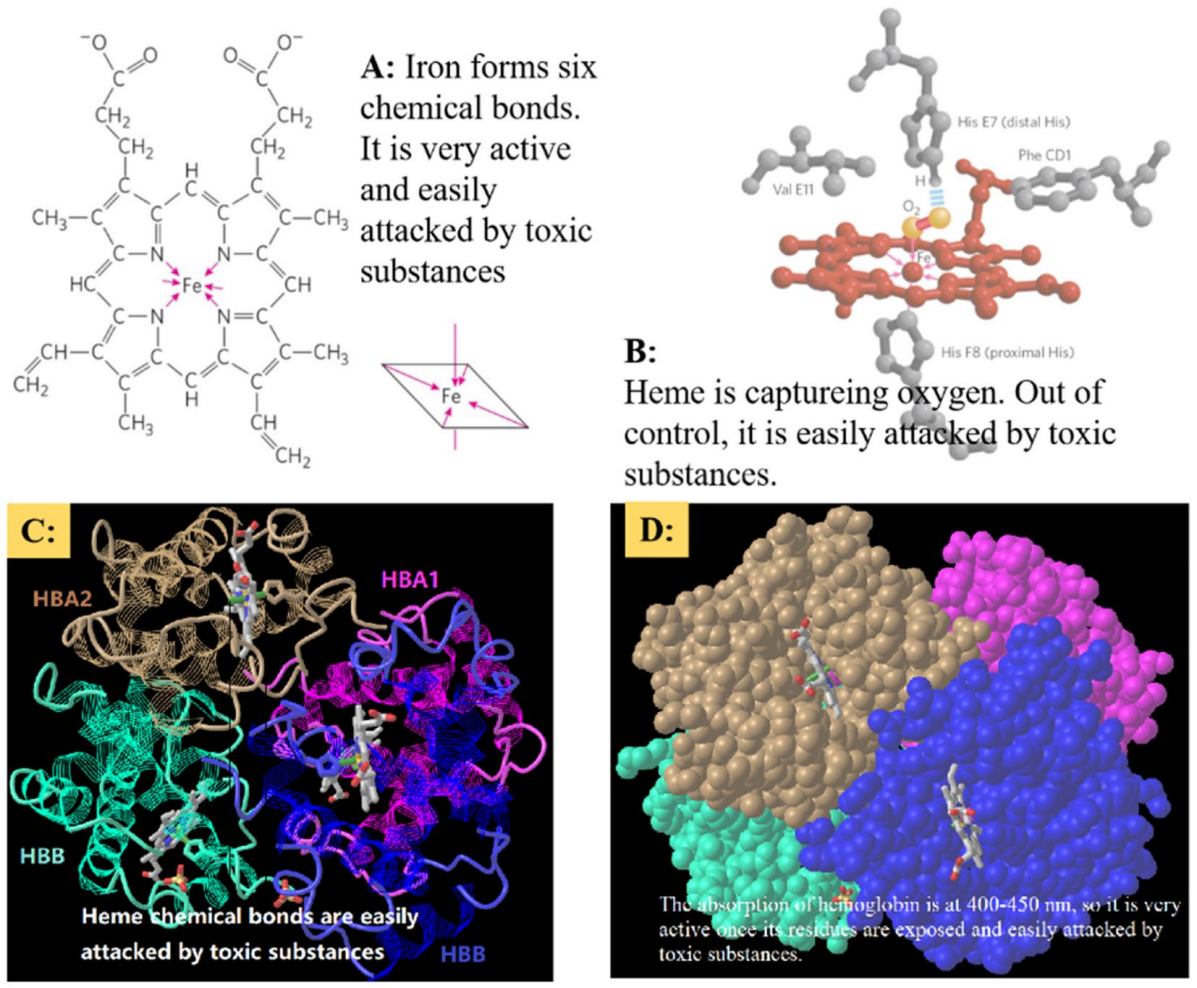
The structure of hemoglobin. (**A**) Heme (iron porphyrin). (**B**) Heme captures oxygen. (**C**) Heme locates in hemoglobin. (**D**) Sphere of Hemoglobin. (Note: Subfigures (**A**, **B**) are downloaded from David et al.^56^ Subfigures (**C**, **D**) are downloaded from website: https://www.ncbi.nlm.nih.gov/Structure/icn3d/full.html?&mmdbid=7599&bu=1&showanno=1&source=full-feature).

To control Fe^2+^ and Fe^3+^, Heme (iron porphyrin) is needed (Fig. 14A). Heme consists of a complex organic ring structure, protoporphyrin IX, featuring a bound iron atom in its ferrous (Fe^2+^) state^56^. The iron atom of heme has six coordination bonds: four in the plane of heme, and bonded to the flat porphyrin ring system, and two are perpendicular to the porphyrin ring (Fig. 14B). And one of the perpendicular coordination bonds to an oxygen molecule, and the other perpendicular coordination is occupied by a His residue^56^. Under the control of Heme, Fe^2+^ combines with oxygen molecules in an orderly manner, and releasing oxygen molecules is ordered too. It should be noted that, because Fe^2+^ is very active, bonding to toxic substances, such as abnormal tau, is very easy, where the infiltration of abnormal tau exists in brain blood as AD occurs because tau is small and holds hydrophilic property (Fig. 13A).

##### The features of haemoglobin

To regulate Heme orderly and send oxygen to other proteins, hemoglobin protein is needed. Four polypeptide subunits are packed together and form a hemoglobin molecule (Fig. 14C). Interactions between the subunits can permit a highly sensitive response to small changes in ligand concentration. Interactions among the subunits in hemoglobin cause conformational changes that alter the affinity of the protein for oxygen. The modulation of oxygen binding allows the O_2_-transport protein to respond to changes in oxygen demand by tissues^56^. In addition, the wavelength of light absorbed by hemoglobin is 400–450 nm, which is a high-energy photon in body. That is, when the residues of hemoglobin are exposed disorderly and its chemical bonds are opened, they are easily combined with toxic substances, such as CO or toxic tau protein.

#### Hypothesis

The above discussion includes three types of features, structure features of NFTs, structure features of hemoglobin, and pathway features of the V–H pathway. The three types of features are related to each other, and their coherence impacts visual information transmission from the retina. Synthesizing the three types of features, the following hypothesis was induced.

As AD occurs, abnormal tau proteins accumulate, some tau proteins package together disorderly and form NFTs, and some penetrate various brain regions. The abnormal tau molecule penetrates brain regions of the V–H pathway. The abnormal tau is more active than CO and O_2_. Then, it combines with the iron of heme of hemoglobin molecule. And this case is similar to CO poisoning, with molecular CO occupying the position of O_2_. So, the hemoglobin expression on the brain regions of the V–H pathway becomes dysregulated, such as down-regulation, and expression disorder (entropy increases). And this dysregulation leads to the disorder of the V–H pathway, such as the correlation among brain regions of the V–H pathway becoming weak, the pathway being blocked, and every region becoming an isolated island. The disorder of the V–H pathway leads to dysfunction of visual information transmission, such as insufficient oxygen supply and insufficient energy supply.

Therefore, the more NFTs, the more abnormal tau existing in the brain regions of V–H pathway, the higher the risk of hemoglobin being attacked by abnormal tau, the more disorder hemoglobin expression, the more discoordination of V–H pathway, the less oxygen supply and energy supply on V–H pathway, the more dysfunctional V–H pathway.

At last, this dysfunction has an impact on the early symptoms of AD, such as spatial orientation disorder and object recognition disorder, where the V–H pathway is responsible for motion and spatial orientation, object recognition, etc.

## Discussion

Alzheimer's disease (AD) is a neurodegenerative disorder characterized primarily by cognitive impairment. Its early symptoms include memory loss, spatial orientation disorder, and object recognition disorder. It is the popular view currently that brain tissue damage involving memory function, such as damage to the hippocampus, is the most common cause of dementia. However, the authors think that the disorder of visual information transmission should have an impact on the symptoms too. So, the conception of the V–H pathway was presented in this paper.

The transmission pathway of visual information is named as Visual–Hippocampal pathway (V–H pathway). It transmits information obtained from the retina to the hippocampus. V–H pathway consists of five brain regions (Fig. 1) and two sub-pathways. One pathway is a dorsal stream, and the other is a ventral stream^33,34^ (Figs. 1 and 2). The dorsal stream primarily is associated with motion perception and spatial localization^35^, while the ventral stream involves higher-level visual functions such as object recognition and face identification ^36^.

**Figure 15:**
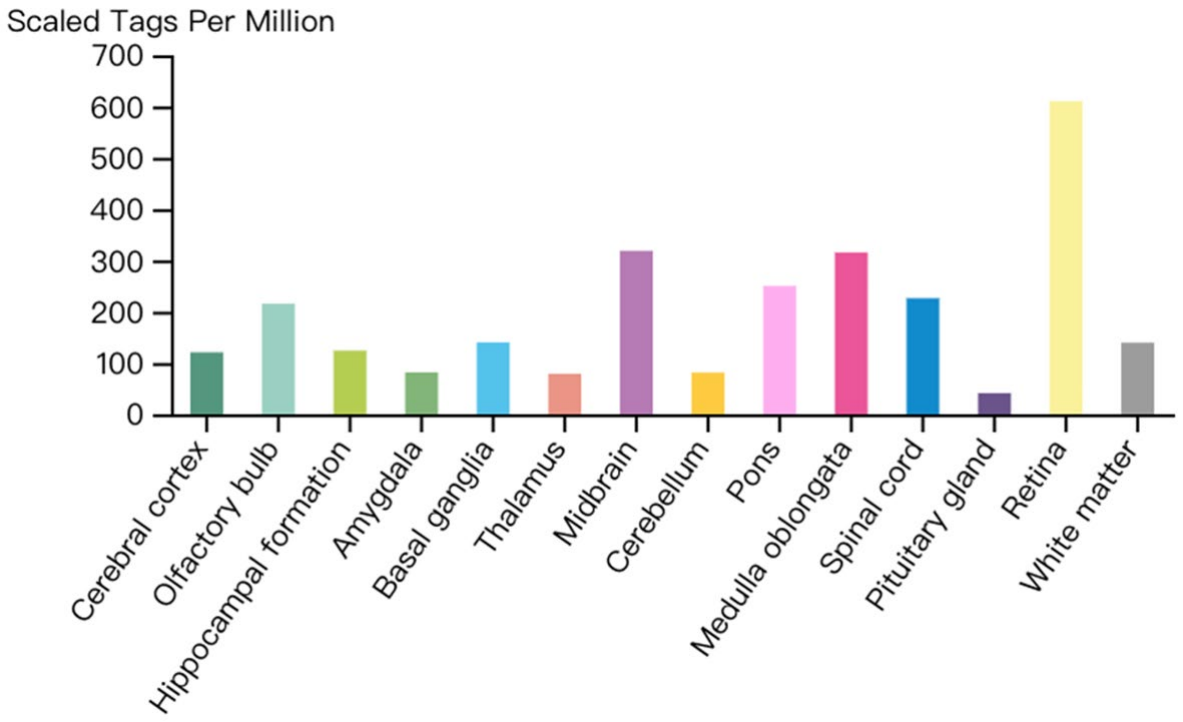
Expression levels of hemoglobin in the FANTOM5 Human Brain CAGE Dataset. The distribution of this figure shows the following feature: Retina holds maximum hemoglobin molecules. And this suggests that, visual information transmission is associated with hemoglobin tightly.

V–H pathway transmits nearly 80% of information, and it should cost a lot of energy. The most of energy-releasing dependents are oxygen and oxygen is carried by hemoglobin. So, by detecting the hemoglobin expression on the V–H pathway, the energy supply of the pathway can be understood. Figure 15 shows that the retinal holds high hemoglobin expression, the V–H pathway needs a large of hemoglobin molecules to generate large energy and support visual information transmission.

Just like CO easily attacks hemoglobin molecules, abnormal tau protein also easily attacks hemoglobin molecules, resulting in the disorder of hemoglobin expression on the V–H pathway and causing pathway dysfunction (Figs. 13 and 14).

As AD occurs, at early stage, toxic substances accumulate. For example, abnormal tau proteins accumulate and form NFTs. Under the attack of toxic substances, hemoglobin expression becomes dysregulated, and the V–H pathway becomes dysfunctional, the following features are generated:

- Hemoglobin expression becomes down-regulated on the brain regions of the V–H pathway (Fig. 7). The capacity of oxygen transport reduces, and the energy supply of the pathway becomes weak.
- Hemoglobin expression on the brain regions of the V–H pathway holds bigger entropy (Fig. 8). Hemoglobin expression becomes more disordered.
- The correlation among different brain regions of the V–H pathway becomes weak (Fig. 9). The coordination of the V–H pathway becomes weak.
- As NFTs increase, the coordination of the V–H pathway reduces gradually and becomes more disordered (Figs. 10, 11, and 12). NFTs increasing suggests that there are more abnormal tau proteins penetrating the bloodstream and the brain regions of the V–H pathway, where the V–H pathway costs needs more bloodstream because it transmits 80% of information. These abnormal tau proteins attack hemoglobin molecules in the brain regions of the V–H pathway. Then hemoglobin on the V–H pathway is dysregulated, and the V–H pathway loses control and becomes disorder.

The dysfunction of the V–H pathway has impacts on early symptoms. The dorsal stream of the V–H sub-pathway is responsible for spatial orientation recognition, and its dysfunction leads to the symptom of spatial orientation recognition disorder. The ventral stream of the sub-pathway is responsible for object recognition, and its dysfunction leads to the symptom of object recognition disorder.

## Materials and methods

### Data collection and preprocessing

The gene chip data used in this study were sourced from the Gene Expression Omnibus (GEO) database, hosted by the National Center for Biotechnology Information (NCBI)^57,58^, with the data accession numbers GSE84422 and GSE1297. The GSE84422 dataset employed sequencing platforms GPL96, GPL97, and GPL570, and comprises 1053 post-mortem brain samples from 19 different brain regions, including the Occipital Visual Cortex, Middle Temporal Gyrus, and Parahippocampal Gyrus. These samples were derived from 125 individuals with varying degrees of dementia and neuropathological severity in Alzheimer's disease. Clinical information, such as the braak neurofibrillary tangle score (Braak) and neurofibrillary tangles (NFTs), are included. The GSE1297 dataset utilized the sequencing platform GPL96 and analyzed hippocampal gene expression in 9 control subjects and 22 AD patients with varying degrees of severity. This dataset includes clinical information such as the mini-mental state examination (MMSE) and neurofibrillary tangles (NFTs). Assuming that the dataset consists of n samples and m genes, we define the gene expression matrix G:

5 $$G=\left[\begin{array}{ccc}{g}_{11}& \cdots & {g}_{1n}\\ \vdots & \ddots & \vdots \\ {g}_{m1}& \cdots & {g}_{mn}\end{array}\right]=\left[\begin{array}{ccc}{\overrightarrow{\alpha }}_{1}& \cdots & {\overrightarrow{\alpha }}_{m}\end{array}\right]={\left({g}_{ij}\right)}_{m\times n}$$

Among them, ${g}_{ij}$ represents the expression level of the $ith$ gene in the $jth$ sample, where each row in the matrix represents a gene, and each column represents a sample. The datasets mentioned above, covering different brain regions and AD disease states, provide the foundation for an in-depth investigation into the gene expression patterns and molecular mechanisms underlying AD-related biological changes.

To reduce experimental errors, samples from the GPL96 platform were used consistently, and the aggregate function was applied to take the median for duplicate genes. Considering the significant variation in gene expression values due to differences in laboratory conditions in microarray experiments, a logarithmic transformation to the base 2 (Formula 6) was applied to the gene expression values in the GSE1297 dataset to convert exponential growth into linear growth. This resulted in the gene expression matrix G', as shown in Formula (7):

6 $${g{\prime}}_{ij}={\mathit{log}}_{2}\left({g}_{ij}+1\right)$$

7 $$G{\prime}={\left({g{\prime}}_{ij}\right)}_{m\times n}$$

After pre-processing, the main variables in the dataset were as follows (Table 1).

**Table 1:** Main variables.

Variable name	Description
Region	Brain region
Category	Neuropathologic classification of AD disease
Braak	Braak neurofibrillary tangle score
NFTs	Sum of neurofibrillary tangles density in multiple brain regions
CDR	Clinical dementia rating
MMSE	Minimal state examination score
HBB	Expression of hemoglobin subunit beta after logarithmically processed
HBA1	Expression of hemoglobin subunit alpha1 after logarithmically processed
HBA2	Expression of hemoglobin subunit alpha2 after logarithmically processed

*MMSE was not included in the GSE84422 dataset.

### Gene expression differential analysis

Gene expression differential analysis, which involves analyzing gene expression levels under different experimental conditions (such as different experimental treatments, patients, or tissue types), is a crucial technique in the analysis of gene chip data. And identifying genes with differential expression is essential for discovering genes related to AD, laying the foundation for AD diagnosis.

The Fold Change (FC) method, which calculates the fold change in gene expression levels between two experimental conditions, is the most widely used technique in gene chip data analysis^59^. It determines the presence of differential genes and the formula is as follows:

8 $$FC=\frac{Condition B Expression}{Condition A Expression},$$

where Condition A represents one condition (e.g., the Normal group in the GSE84422 dataset), and Condition B represents another condition (e.g., the Possible ad/Probable ad/definite ad groups in the GSE84422 dataset). The advantage of the fold change analysis lies in its cost-effectiveness and simplicity. However, it lacks a reasonable threshold for selecting differentially expressed genes, making it sensitive to genes with significant fold changes and less reliable for genes with small fold changes. Therefore, FC is generally used for preliminary experiments or initial screening.

Limma (Linear models for microarray analysis) is a Bioconductor package that provides a comprehensive solution for the analysis of gene expression experimental data^60^. It improves the accuracy and reliability of data analysis by integrating various statistical principles, helping researchers better understand the complexity and diversity of gene expression data. Limma has become a popular choice for differential expression analysis of microarray data in the past decade. To analyze gene expression differences, Limma uses linear models to estimate the significance of differential expression. It fits a linear model to the expression data for each gene and then performs statistical tests on the model parameters to determine which genes are significantly differentially expressed.

In this study, the fold change method is used to analyze gene expression changes between the Normal group and the Possible ad/Probable ad/definite ad groups in the GSE84422 dataset. Genes with fold change values greater than 1.5 and less than 0.06 were selected. Furthermore, the same processing was applied to the Control group and the Incipient/Moderate/Severe groups in the GSE1297 dataset. The intersection of the results from both methods was used to identify key genes associated with AD.

Simultaneously, the Limma package in R was employed for gene selection. And the aggregate function was used to preprocess the data. Subsequently, the lmFit function was applied to perform differential analysis between the Normal group and the Possible ad/Probable ad/definite ad groups in the GSE84422 dataset, filtering genes with |logFC| ≥ 0.5 and p-value < 0.05. Additionally, the same procedure was applied to the Control group and the Incipient/Moderate/Severe groups in the GSE1297 dataset. The results from both datasets were intersected, serving as an initial determination of key genes associated with AD pathology. Finally, the intersection of the results from both the fold change and Limma analyses identified the key genes (HBB, HBA1, HBA2) associated with AD.

### Protein–protein interaction (PPI) network analysis

After selecting key genes using the Limma and fold change methods, the results from the two datasets were merged. Subsequently, utilizing the STRING website, PPI analysis was performed on the identified key genes to acquire protein–protein interaction information^61^. Afterward, the Cytoscape software was employed to generate the PPI network.

### Pearson correlation coefficient matrix

The Pearson correlation coefficient is used to measure the degree of linear correlation between variables and can reflect whether genes are highly correlated. The calculation formula is as follows:

9 $$r=\frac{\sum ({x}_{i}-\overline{x })({y}_{i}-\overline{y })}{\sqrt{\sum {\left({x}_{i}-\overline{x }\right)}^{2}\sum {\left({y}_{i}-\overline{y }\right)}^{2}}}$$

To investigate the coordination of the three hemoglobin genes (HBB, HBA1, HBA2) in the Visual–Hippocampal brain pathway, the Pearson correlation coefficient was used to study the correlation of HBB, HBA1, and HBA2 expression levels in different brain regions and stages of AD. Using the AD stage (Normal, Possible ad, Probable ad, Definite ad) as the division criterion, the GSE84422 dataset was divided into four subsets,${G{\prime}}_{Normal}$, ${G{\prime}}_{Possible ad}$, ${G{\prime}}_{Probable ad}$, ${G{\prime}}_{Definite ad}$, as the original inputs. The correlation coefficient matrix was obtained through the following calculation process:

**Figure:**
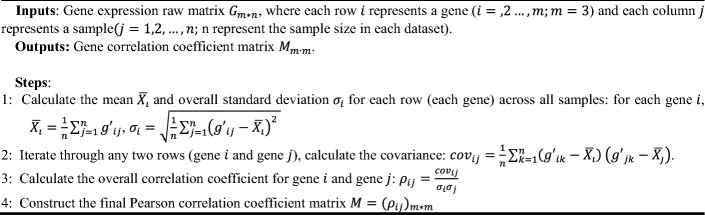
**Algorithm 1**. Pearson correlation coefficient matrix.

Four matrices of the correlation coefficients were then obtained as follows: ${M}_{Normal}$, ${M}_{Possible ad}$, ${M}_{Probable ad}$, ${M}_{Definite ad}$.

### Entropy

#### Boltzmann entropy

The intuitive understanding of entropy is Boltzmann's formula:

10 $${S}_{Boltzmann}=klnW,$$

where $W$ denotes the number of microscopic states and $k$ denotes Boltzmann constant, and ${\text{S}}_{\text{Boltzmann}}$ is the entropy of the system of uniform distribution.

Let $p=1/w$. That is, $p$ is the probability of uniform distribution. Then, we have

11 $${S}_{Boltzmann}=kln\frac{1}{p}=-klnp$$

The bigger $W$, the smaller $p$, the bigger ${\text{S}}_{\text{Boltzmann}}$, the more disorder the system. So, Boltzmann entropy is the measurement of system chaos.

#### Gibbs entropy (Clausius entropy)

For the system holds $n$ different types of states, according to Eq. (11), its entropy is

12 $$S=E\left\{-kln{(p}_{i})\right\}=-k\sum_{i=1}^{n}{p}_{i}ln{(p}_{i}),$$

where ${p}_{i}$ denotes the probability of the $i$th state.

In the paper, Gibbs free energy is analyzed, the entropy $\Delta H$ included in the formula $\Delta G=\Delta H-T\cdot \Delta S$ refers to Gibbs entropy. Gibbs entropy is defined as

13 $${S}_{Gibbs}=-k\sum_{i=1}^{n}{p}_{i}ln{(p}_{i})$$

When a system tends towards equilibrium, Gibbs entropy does degenerate into Boltzmann entropy.

#### Shannon Entropy

Shannon Entropy is defined by Shannon as

14 $${S}_{Shannon}=-\sum_{i=1}^{n}{p}_{i}{log}_{2}{(p}_{i})$$

15 $${S}_{Gibbs}=(k\frac{1}{{{log}_{2}}^{e}}){S}_{Shannon}$$

Gibbs entropy and Shannon entropy are essentially the same, as they differ by only a constant. Therefore, Shannon entropy is calculated and used it to replace Gibbs entropy in this paper.

#### Calculation of Shannon entropy

Hemoglobin consists of three subunits HBB, HBA1, HBA2. Calculate the correlation coefficient matrix among the subunits. Then induce the eigenvalues of the matrix. At last, calculate entropy from the eigenvalues. The detail of the calculation is listed in the following table.

**Figure:**
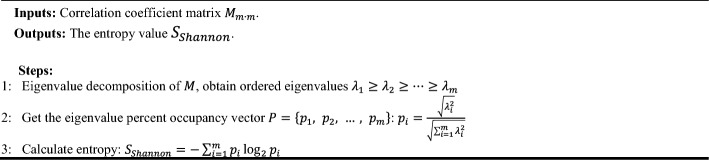
**Algorithm 2**. Entropy calculation.

### Cosine similarity, inner product, and magnitude product

**Figure 16:**
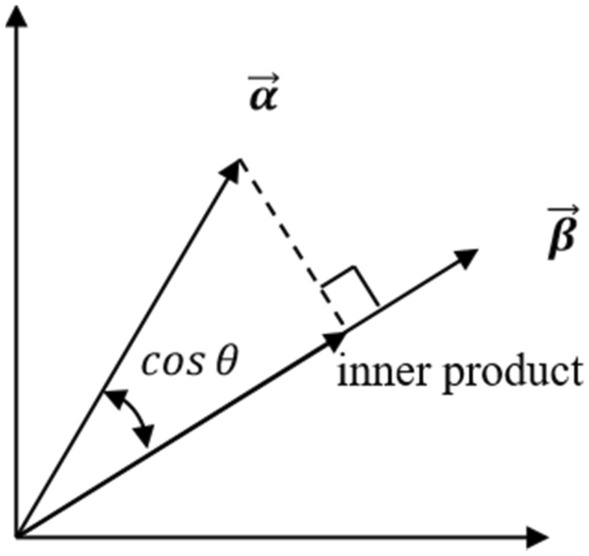
Vector Cosine Similarity and Inner Product Schematic.

Cosine similarity ($\text{cos}\theta$) measures the cosine value of the angle between two vectors in space and is used to assess their directional similarity, as illustrated in Fig. 16. A smaller angle indicates greater vector similarity, and cosine similarity approaches 1 or − 1 (representing identical or opposite directions, respectively). We took the expression levels of the three genes HBB, HBA1, and HBA2, represented by the variables HBB, HBA1, and HBA2 in the dataset, to form a triplet $\left(\text{HBB},\text{ HBA}1,\text{ HBA}2\right)$. Then, a three-dimensional vector $\overrightarrow{\chi }=\left(\text{HBB},\text{ HBA}1,\text{ HBA}2\right)=\left({x}_{1}, {x}_{2},{x}_{3}\right)$, representing the gene expression vector of HBB, HBA1, and HBA2 in given brain region, was defined to measure the similarity in gene expression trends between different samples using cosine similarity.

Cosine similarity calculation involves vector inner product and magnitude product. The inner product of vectors (inner product), also known as the scalar product, is the sum of the products of corresponding components of vectors. It calculates the projection of one vector onto another's direction, as illustrated in Fig. 16. In the context of this paper, the inner product between gene expression vectors represents the overall similarity between samples, including expression strength and direction. The magnitude of a vector, which is the distance from the origin to the tip of the vector, can be calculated using different norms, typically using the ${{\ell}}_{2}$ norm. The magnitude of sample gene expression vectors can measure the expression strength of genes.

To study the relationship between hemoglobin expression levels in different groups (Normal/Possible ad/Probable ad) of the GSE84422 dataset and clinical NFT, we calculate the magnitude product, inner product, and cosine similarity of gene expression vectors corresponding to different brain regions for each group.

**Figure:**
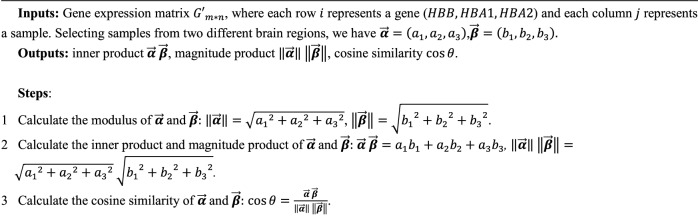
**Algorithm 3**. Inner product, magnitude product, cosine similarity.

## Conclusion

AD is a neurodegenerative disorder characterized primarily by cognitive impairment. Its early symptoms include memory loss, spatial orientation disorder, and object recognition disorder.

The motivation of this paper is to explore the features of the visual information transmission pathway (V–H pathway) when AD occurs. The following features were observed:

- Hemoglobin expression on the brain regions of the V–H pathway is abnormal as AD occurs, and it is down-regulated.
- Hemoglobin expression becomes a disorder in the brain regions of the V–H pathway.
- The coordination of the V–H pathway becomes weak as NFTs increase, and the pathway becomes more dysfunctional.

According to the features, the following hypothesis was proposed.

As NFTs increases, more and more abnormal tau proteins penetrate the bloodstream and arrive at the brain regions of the V–H pathway. The hemoglobin molecules have a higher risk of attacking by abnormal tau proteins or other toxic substances. Under the attack of toxic substances, hemoglobin expression becomes more dysregulated, then the energy supply to the V–H pathway becomes disordered and the V–H pathway becomes dysfunctional. This dysfunction has an impact on early symptoms of AD, such as spatial recognition disorder and face recognition disorder.

## Data Availability

Sequence data that support the findings of this study have been deposited in the National Center for Biotechnology Information with the primary accession code GSE84422 and GSE1297 (https://www.ncbi.nlm.nih.gov/geo/, accessed on 7 June 2023).
